# Plant-inspired adhesive and tough hydrogel based on Ag-Lignin nanoparticles-triggered dynamic redox catechol chemistry

**DOI:** 10.1038/s41467-019-09351-2

**Published:** 2019-04-02

**Authors:** Donglin Gan, Wensi Xing, Lili Jiang, Ju Fang, Cancan Zhao, Fuzeng Ren, Liming Fang, Kefeng Wang, Xiong Lu

**Affiliations:** 1 Key Lab of Advanced Technologies of Materials, Ministry of Education, School of Materials Science and Engineering, Southwest Jiaotong University, 610031 Chengdu, Sichuan China; 2 Key Laboratory of Fluid and Power Machinery of Ministry of Education, School of Materials Science and Engineering, Xihua University, 610039 Chengdu, China; 3 Department of Materials Science and Engineering, Southern University of Science and Technology, Shenzhen, 518055 Guangdong, China; 4 Department of Polymer Science and Engineering, School of Materials Science and Engineering, South China University of Technology, Guangzhou, 510541 China; 5 National Engineering Research Center for Biomaterials, Sichuan University, 610064 Chengdu, China

## Abstract

Adhesive hydrogels have gained popularity in biomedical applications, however, traditional adhesive hydrogels often exhibit short-term adhesiveness, poor mechanical properties and lack of antibacterial ability. Here, a plant-inspired adhesive hydrogel has been developed based on Ag-Lignin nanoparticles (NPs)triggered dynamic redox catechol chemistry. Ag-Lignin NPs construct the dynamic catechol redox system, which creates long-lasting reductive-oxidative environment inner hydrogel networks. This redox system, generating catechol groups continuously, endows the hydrogel with long-term and repeatable adhesiveness. Furthermore, Ag-Lignin NPs generate free radicals and trigger self-gelation of the hydrogel under ambient environment. This hydrogel presents high toughness for the existence of covalent and non-covalent interaction in the hydrogel networks. The hydrogel also possesses good cell affinity and high antibacterial activity due to the catechol groups and bactericidal ability of Ag-Lignin NPs. This study proposes a strategy to design tough and adhesive hydrogels based on dynamic plant catechol chemistry.

## Abstract (web summary)

Biomimetic catechol-based adhesives have attracted significant interest but can lose adhesion due to excessive oxidation. Here, the authors report on the addition of silver-Lignin nanoparticles as a dynamic catechol redox system to maintain catechol/quinone balance, making a reusable, antibacterial bioadhesive.

## Introduction

Hydrogels have drawn attention as ideal materials for the repair of soft tissue, including skin^1, 2^, cartilage^3^, and muscle^4^. However, the poor mechanical properties of hydrogels have limited their application in biomedical engineering. Various approaches have been used to prepare hydrogels with excellent mechanical properties, such as nanocomposite hydrogels^5^, topological hydrogels^6^, and double-network hydrogels^7^. However, many tough hydrogels possess poor cell or tissue adhesion, which preclude the integration of the hydrogel with human tissues. Recently, adhesive hydrogels have been designed based on different mechanism. For example, an adhesive hydrogel consisting of an adhesive surface and an energy dissipative matrix has been prepared^8^. Another adhesive hydrogel tackified by a nucleobase exhibits good adhesive properties because the nucleobase forms adhesive interactions with various materials^9^. Sundew-inspired adhesive hydrogels have been developed because the hydrogel has a well-patterned scaffold structure similar to the structure of sundew mucilage^10^.

Recently, catechol chemistry has shed light on a method for preparing a hydrogel with good adhesiveness and super toughness^11^. Mussel-inspired adhesive hydrogels with polydopamine (PDA) are typical examples of catechol-chemistry-based adhesive hydrogels^12–14^. In these hydrogels, the catechol functional groups of PDA form covalent bonds/noncovalent bonds with different materials, and therefore these hydrogels exhibit good adhesion to various surfaces^15^. Nevertheless, pure PDA-functionalized hydrogels generally have poor mechanical properties^16–18^ and these adhesive hydrogels are not reusable^19^. This is because some of the catechol groups in the adhesive hydrogel are converted to quinone groups through oxidation. The catechol groups can form physical or chemical bonds with different surfaces and the quinone groups promote cohesion in the hydrogel. However, overoxidation of catechol groups cause loss of adhesiveness of the hydrogel^20–22^. Actually, mussel retains its long-term adhesion because a reductive protein is continuously secreted in mussel footprints to maintain a dynamic balance between the quinone and catechol groups, which allows the retention of long-term adhesion^23^. The adhesion properties of mussels inspired us to consider that controlling the oxidation degree of catechol groups is vital to maintaining the adhesive features of PDA-containing hydrogels. In our recent study, we controlled the oxidation process of the catechol groups and maintained the quantity of catechol groups in the hydrogel to obtain tough hydrogels with excellent repeatable and durable adhesiveness^24, 25^.

Many plants have inherent adhesiveness because extracellular matrix of plants contain adhesive molecules, although plant-based adhesives are brittle and vulnerable^26, 27^. Lignin is the second-most abundant biopolymer originating from plants and has been widely used in biomedical engineering owing to its biocompatibility and environmental friendliness^28–30^. Lignin possesses multiple functional groups such as reductive phenolic hydroxyls and methoxy groups, which can serve as reducing and stabilizing agents^31^. Lignin has been used as a phenolic resin adhesive because of the reactive functional groups^32, 33^. It should be noted that the lignin-based adhesiveness in most previous reports are short-term and non-repeatable^34, 35^. The phenolic hydroxyls or methoxy groups on the lignin can be converted to redox-active quinone/hydroquinone, and consequently to semiquinone radicals by a comproportionation reaction^36^. The phenol or methoxy groups in lignin can reduce silver ions (Ag^+^) to metallic silver nanoparticles (Ag NPs)^37^, and these functional groups were oxidized to the corresponding quinone/hydroquinone in this process. Furthermore, the Ag NPs can form photogenerated electrons because of surface plasmon resonance^38, 39^. In the presence of the photogenerated electron, the quinone/hydroquinone groups in lignin were converted into catechol groups^40, 41^. The previous studies inspired us to propose a long-lasting adhesive hydrogel based on plant catechol chemistry.

Inspired by plants, a catechol-chemistry-based hydrogel with long-term adhesiveness, high toughness, and antibacterial ability is developed. The hydrogel is gelled from an aqueous precursor solution containing Ag-Lignin NPs, pectin, and acrylic acid (AA) under an ambient environment. Pectin and polyacrylic acid (PAA) forms an interpenetrating network with multi-crosslinking of covalent bonds and noncovalent bonds, which endows the hydrogel with excellent mechanical properties. The hydrogel displays long-term and repeatable adhesiveness because the Ag-Lignin NPs continuously generate the catechol groups in the inner hydrogel network. The hydrogel is spontaneously polymerized under room temperature conditions because Ag-Lignin NPs interact with ammonium persulfate (APS) to produce a large amount of free radicals and initiate the polymerization of the hydrogel. Consequently, ultraviolet (UV) or thermal treatment, which is harmful to cells/tissues, is avoided. Because of the convenient direct gelation, this plant catechol-chemistry-based adhesive and tough hydrogel is suitable for surgical operation or other biomedical applications.

## Results

### Design rationale of the hydrogel

**Fig. 1:**
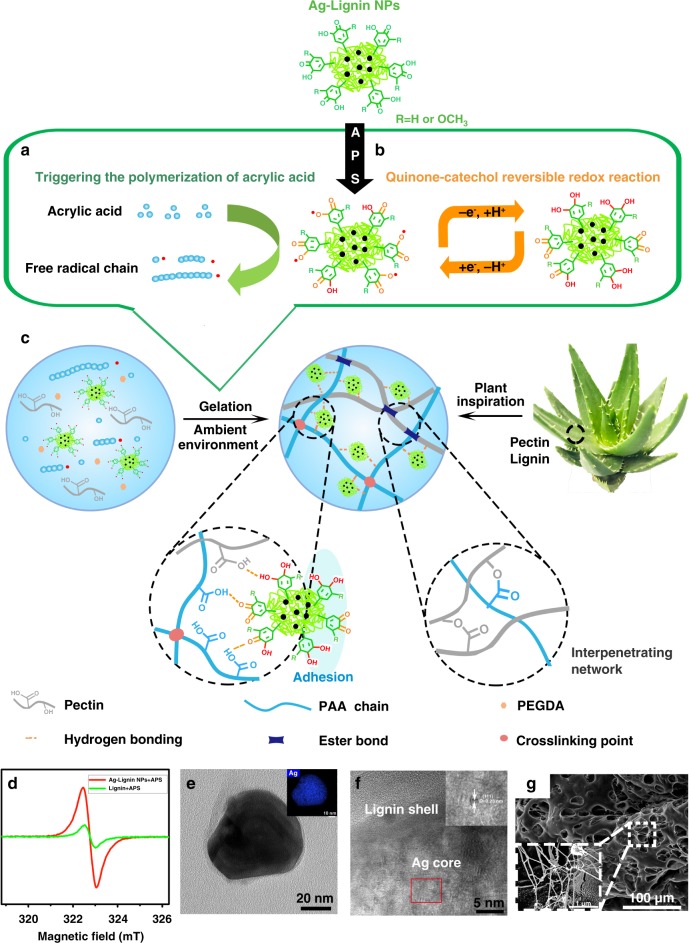
Design strategy for the plant-inspired catechol-chemistry-based self-adhesive, tough, and antibacterial NPs-P-PAA hydrogel. **a** Generation of radicals by the redox reaction between Ag-Lignin NPs and ammonium persulfate (APS), triggering the gelation of the hydrogel under an ambient environment. **b** Quinone-catechol reversible reaction maintains dynamic balance. **c** Scheme of molecular structure of plant-inspired adhesive and tough hydrogel. **d** Electron spin-resonance spectroscopy (ESR) spectra for quinone radical detection. **e** Transmission electron microscope (TEM) micrograph shows the core-shell structure of Ag-Lignin NPs; the inset is Ag element mapping. **f** High-resolution transmission electron microscopy (HRTEM) micrograph shows the structure of Ag-Lignin NPs; the inset is high-resolution lattice. **g** Scanning electron microscope (SEM) micrograph shows the microfibril structures in the hydrogel; the inset presents typical microfibrils. NPs, nanoparticles; P, pectin; PAA, polyacrylic acid

The Ag-Lignin NPs-PAA-pectin hydrogel was prepared in two steps, as shown in Fig. 1. First, Ag-Lignin NPs core-shell nanostructures were synthesized through a redox reaction between lignin and the [Ag(NH_3_)_2_]^+^ complex (Supplementary Figure 1). During this process, the methoxyl and catechol groups of lignin are oxidized into quinone groups, as proved by cyclic voltammetry (CV) experiments, Fourier-transform infrared spectroscopy, and X-ray photoelectron spectroscopy (XPS) analysis (Supplementary Figure 5, 6, 7 and 8, Supplementary Table 4, and Supplementary Notes 7–9). Second, AA monomers and pectin were mixed with the Ag-Lignin NPs suspension to form the Ag-Lignin NPs-PAA/pectin nanocomposite hydrogel owing to the Ag-Lignin NPs-triggered free-radical polymerization under an ambient environment (Fig. 1a). The Ag-Lignin NPs have the ability to realize the quinone-catechol reversible reaction, which endows the hydrogel with adhesiveness (Fig. 1b).

In this hydrogel, both the lignin and pectin originated from plants. Pectin is a heteropolysaccharide, and is the main component in the primary cell walls of plants. Pectin contains -OH and -COOH groups, which act as active sites for hydrogen bonds or ionic bonds. These pectin functional groups provide selective and strong bonding with certain substances^42^. Moreover, pectin improved the biocompatibility and also interpenetrated into the PAA network to make the hydrogel tougher and more flexible (Fig. 1c). Lignin formed Ag-Lignin NPs to create a redox environment inside the hydrogel network, which was the key factor for the preparation of such a multifunctional hydrogel. First, Ag-Lignin NPs can construct the dynamic catechol redox system inner hydrogel network, which mimics the long-lasting reductive/oxidative environment in mussel footprint and continuously generates catechol groups, and therefore endows the hydrogel with repeatable and long-lasting adhesion ability. As shown in Fig. 1b, the quinone-catechol reversible reaction of Ag-Lignin NPs maintains a dynamic balance inside the aqueous hydrogel network with the assistance of free electrons from surface plasmon resonance of Ag NPs (Supplementary Notes 10 and Supplementary Figure 9) and the H^+^ from ionization of PAA, which continuously replenishes the catechol and quinone groups in the hydrogel. Second, Ag-Lignin NPs generate free radicals that trigger self-gelation of the hydrogel. Therefore, the hydrogel was able to cure under an ambient environment. As shown in Supplementary Figure 1, under an alkaline environment, the functional groups, such as -OCH_3_ or -OH on lignin, are oxidized by silver ions to quinone/semiquinone free radicals. After the addition of APS, large amounts of free radicals are continuously generated to initiate steady free-radical polymerization (Fig. 1a). Therefore, UV irradiation or thermal initiation with toxic auxiliary agents, such as tetramethylethylenediamine, was avoided during hydrogel gelation. Finally, the functional groups of Ag-Lignin NPs form noncovalent interaction with the PAA and pectin, and therefore the NPs work as nano-reinforcement to improve the mechanical properties of the hydrogel (Fig. 1c).

To further investigate the mechanism of the free radical generated by Ag-Lignin NPs, electron spin-resonance spectroscopy (ESR) was used to characterize free radicals in various solutions (Fig. 1d, Supplementary Figure 1, 2 and Supplementary Table 2). The ESR spectrum of the Ag-Lignin NPs with APS contained a signal with a *g* value of 2.0034, which was attributed to quinone/semiquinone radicals^36^. The ESR spectrum of the pure lignin/APS solution exhibited the same peak with a lower intensity, which indicated that the quantity of the quinone/semiquinone radicals was much smaller (Supplementary Table 3). These results demonstrated that pure lignin or APS solution can generate free radicals, but the quantity of free radicals in the solution is not enough to trigger free-radical polymerization. Thus, only the Ag-Lignin NPs-APS solution can generate enough radicals to trigger the self-polymerization of the hydrogel under an ambient environment, whereas lignin or APS alone did not (Supplementary Figure 10). Furthermore, Ag-Lignin NPs-APS solution can initiate the polymerization of other free-radical monomers, such as AA, acrylamide, and poly(ethylene glycol) diacrylate (Supplementary Figure 11).

Scanning electron microscope (SEM) and transmission electron microscope (TEM) micrographs revealed that the Ag-Lignin NPs formed clusters with core-shell structures (Fig. 1e, f and Supplementary Figure 3). The DLS analysis showed that the average size of the Ag-Lignin NPs was 125∼145 nm (Supplementary Figure 4). After incorporation in the hydrogel, the Ag-Lignin NPs were well dispersed in the network (Fig. 1g). The distribution of the Ag-Lignin NPs in the hydrogel was also revealed by element mapping of Ag, and the results indicated that Ag was distributed uniformly inside the hydrogel (Supplementary Figure 17). With the presence of catechol groups of Ag-Lignin NPs, the freeze-dried hydrogel exhibited an interwoven microfibril structure. The microfibrils were a distinctive feature that only present in the NPs-P-PAA hydrogel, but not in pure PAA and P-PAA hydrogels (Supplementary Figure 16a, b). The microfibrils were formed by the interactions between polymer chains and NPs because catechol groups of Ag-Lignin NPs can form intermolecular interactions with the polymer chains and generate nanostructured morphologies^43^.

### Dynamic reductive/oxidative reaction in the NPs-P-PAA hydrogel

The redox environment inner the NPs-P-PAA hydrogel was investigated by XPS analysis and CV experiments (Supplementary Notes 13, 14). XPS analysis was used to investigate changes in the content of catechol group during the redox reaction. The XPS results indicated that lignin had high contents of C-O and C-OH groups at 285.9 eV and a low content of C = O at 288.6 eV. For the Ag-Lignin NPs, the C 1*s* spectrum showed that the contents of C-O and C-OH groups sharply decreased and the content of C = O groups greatly increased (Supplementary Figure 8). These changes were evidence of oxidation and the associated reaction between lignin and [Ag(NH_3_)_2_]^+^. In the NPs-P-PAA hydrogel, both C-O(C-OH) and C = O appeared, which indicated that C-O or C-OH (catechol) groups were present in the NPs-P-PAA hydrogel (Supplementary Figure 12 and Supplementary Table 6). CV experiments were conducted to investigate the redox reaction in the NPs-P-PAA hydrogel system. The curves of CV scanning presented a prominent redox peak at 0.10∼0.20 V (Supplementary Figure 13a), which corresponded to catechol oxidation and quinone reduction that occurred at the same potential^44, 45^. These redox peaks still existed even with excessive persulfate (Supplementary Table 7 and Supplementary Figure 13b–e), which indicated that the stable redox reaction of Ag-Lignin NPs happened even in excessive persulfate solution. In short, both the results of XPS analysis and CV experiments prove that redox exchange occurs in the hydrogel. Furthermore, the antioxidative abilities of the hydrogels were tested by measuring their capacities to scavenge α, α-diphenyl-β-picrylhydrazyl (DPPH) free radicals, and the results indicate that both the NPs and NPs-P-PAA hydrogels have high reduction abilities (Supplementary Figure 14, Supplementary Table 8, and Supplementary Notes 15).

### Mechanical properties

**Fig. 2:**
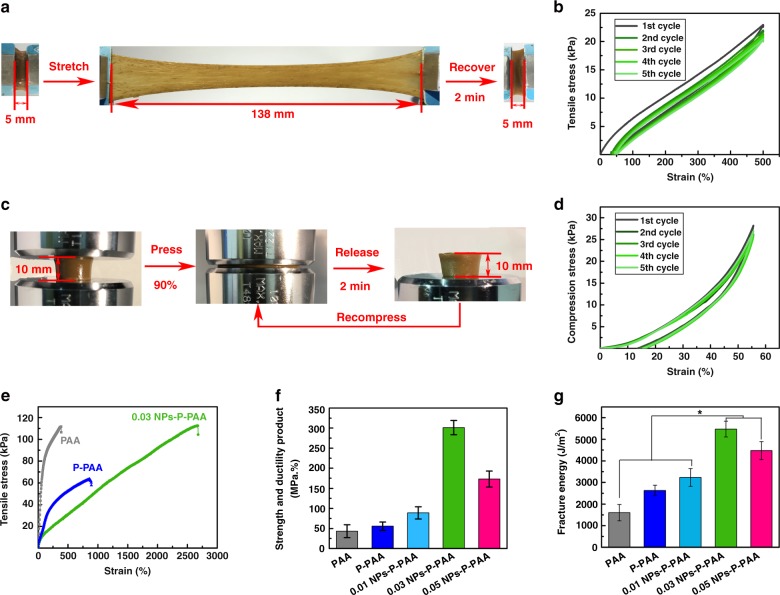
Mechanical properties of the hydrogels. **a** The 0.03 NPs-P-PAA hydrogel was elongated to 26 times its initial length and recovered in 2 min. **b** Tensile loading–unloading curves of 0.03 NPs-P-PAA hydrogel. **c** The 0.03 NPs-P-PAA hydrogel was compressed and recovered in 2 min. **d** Compressive loading–unloading curves of 0.03 NPs-P-PAA hydrogel. **e** Typical tensile stress–strain curves of the hydrogel. **f** Strength and ductility product of various hydrogels. **g** Fracture energy of the hydrogels. (Error bar means the standard deviation, *indicates statistically difference at *p* < 0.05, *p* value was generated by one‐way analysis of variance (ANOVA), followed by Tukey's multiple‐comparison post hoc test, *n* = 4.) NPs, nanoparticles; P, pectin; PAA, polyacrylic acid

The NPs-P-PAA hydrogel was resilient, stretchable, and tough. As shown in Fig. 2a, the hydrogel was stretched to 26 times its initial length. The load–unload tensile stress–strain curves proved it as well (Fig. 2b). It also withstood a high compression to complete deformation and did not break; after the compressive load was removed, the hydrogel recovered automatically and rapidly to its initial shape (Fig. 2c). The load–unload compression stress–strain curves indicated that the NPs-P-PAA hydrogel have good recoverability (Fig. 2d and Supplementary Figure 19). Figure 2e shows the typical tensile stress–strain curves of the hydrogels under tensile tests. The maximum tensile strain increased with the content of NPs and reached a maximum value of 2660% at the 0.03 NPs, much higher than that of the P-PAA hydrogel (860%), and in sharp contrast to that of the PAA hydrogel (380%). The strength and ductility product of various hydrogels was also measured and the 0.03 NPs-P-PAA hydrogel exhibited the highest value (300 MPa%) shown in Fig. 2f. The fracture energy showed a similar trend, as demonstrated by the single edge notched tests (Fig. 2g). A maximum fracture energy of 5500 J m^−2^ was achieved at 0.03 NPs, which was much larger than that of human skin (∼2000 J m^−2^). The high toughness and good resilience of the hydrogel was attributed to two factors. First, pectin interpenetrated the PAA networks and strengthened the hydrogel. Second, the Ag-Lignin NPs, PAA, and pectin had noncovalent interactions between each other, which dissipated energy under large deformation and improved the mechanical properties of the hydrogel. Third, the Ag-Lignin NPs have many hydrophilic functional groups, and therefore can be well dispersed in the hydrogel. Thus, the mechanical properties of the hydrogel were improved because of the nano-reinforcement effects of the NPs. As a control, pure lignin was incorporated in the L-P-PAA hydrogel, and the tensile strength of the L-P-PAA hydrogel was lower than those of the PAA, P-PAA, and 0.03 NPs-P-PAA hydrogels. This is because lignin has many hydrophobic methoxy groups and therefore cannot be uniformly distributed in the hydrogel, demonstrating that bare lignin cannot improve the mechanical properties of the hydrogel (Supplementary Figure 18).

### Adhesiveness

**Fig. 3:**
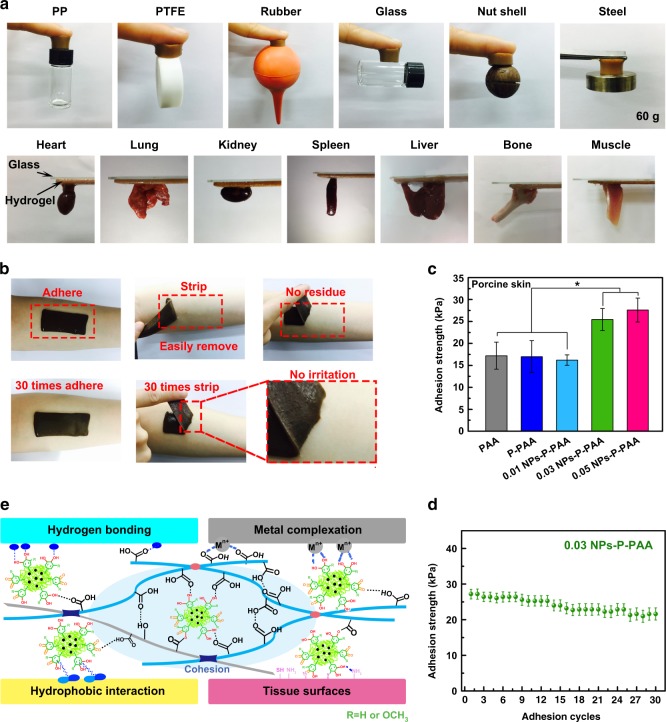
Adhesive properties of the NPs-P-PAA hydrogel. **a** The hydrogel was adhered to various material surfaces and tissues. **b** The hydrogel was repeatedly adhered on the skin of the author. After peeling off, no residue or irritation on skin was found. **c** The adhesive strength of various hydrogels to porcine skin. **d** The repeated adhesion of 0.03 NPs-P-PAA hydrogels to porcine skin after 30 cycles of adhering–stripping. **e** The adhesion mechanism of the NPs-P-PAA hydrogel. The blue oval indicated the hydrogen bonding or hydrophobic interaction between the hydrogel and different surfaces. (Error bar means the standard deviation, * indicates statistically difference at *p* < 0.05, *p* value was generated by one‐way analysis of variance (ANOVA), followed by Tukey's multiple‐comparison post hoc test, *n* = 4.) NPs, nanoparticles; P, pectin; PAA, polyacrylic acid

Similar to an adhesive plant, this hydrogel had long-term and repeatable adhesiveness to a variety of substrates. The hydrogels can adhere to both hydrophilic and hydrophobic surfaces, such as polypropylene, Teflon (PTFE), rubber, glass, nut shell, and steel (Fig. 3a). The adhesive hydrogels also had high adhesiveness to biological tissue, including heart, lung, kidney, spleen, liver, bone, and muscle, which is crucial for biomedical applications (Fig. 3a). The hydrogel exhibited excellent adhesive performance on the skin surface of the author’s body and was peeled off without any residue and anaphylactic reaction (Fig. 3b). The adhesion strength of the hydrogel on representative surfaces was quantified by a tensile adhesion test (Fig. 3c and Supplementary Figure 20); the adhesion strength to glass, titanium (Ti), PTFE, and porcine skin was 38, 50, 65, and 27.5 kPa, respectively. The hydrogel maintained good adhesion even after 30 repeated peeling/adhering cycles (Fig. 3d). To prove that the hydrogels had long-lasting adhesiveness, tensile adhesion testing was performed on NPs-P-PAA hydrogels with different storage times (7, 14, and 28 days). The hydrogel maintained good adhesion to porcine skin after 28 days (Supplementary Figure 21).

Thus, the adhesiveness of the hydrogel was attributed to the synergistic effect of the carboxyl groups of PAA and catechol groups of Ag-Lignin NPs (Supplementary Notes 16 and Supplementary Figure 15). First, the carboxyl groups of PAA can interact with various surfaces through electrostatic interactions. Second, Ag-Lignin NPs generate catechol and quinone groups during the redox reaction, which endows the hydrogel with adhesiveness. Quinone groups form physical crosslinking with the pectin and PAA, which can dramatically enhance the cohesion of the hydrogel. Catechol groups possess strong adhesion to various substrates, which interact with different substrates through covalent and noncovalent bonding (Fig. 3e). Covalent bonding was formed at some specific substrates containing amine or thiol groups through Schiff base or Michael addition reactions; noncovalent bonding, such as hydrogen bonding, π-π stacking, and metal coordination or chelating, could also exist between hydrogels and solid surfaces^43^. Two types of hydrogels were tested to investigate the synergistic effects of the carboxyl groups of PAA and catechol groups of the Ag-Lignin NPs on the adhesiveness of the hydrogel. A hydrogel without carboxyl groups was prepared from polyacrylamide, pectin, and Ag-Lignin NPs (NPs-P-PAM gel), and a hydrogel with low content of carboxyl groups were prepared from poly(acrylic acid-co-acrylamide), pectin, and Ag-Lignin NPs (NPs-P-P(AA-co-AM) gel) (Supplementary Notes 21 and Supplementary Table 9). As shown in Supplementary Figure 22, the adhesion strengths of NPs-P-PAM and NPs-P-P(AA-co-AM) hydrogels to porcine skin were 12 kPa and 15 kPa, respectively, which were lower than that of the NP-P-PAA hydrogel (25 kPa). These results proved that both carboxyl groups of PAA and catechol groups of Ag-Lignin NPs contributed to the good adhesiveness of the hydrogel.

### Antibacterial activity

**Fig. 4:**
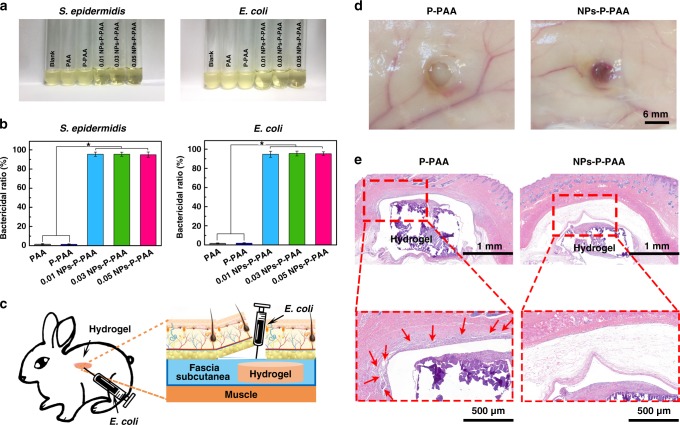
The antibacterial activity of the hydrogel. **a** Photos of *S. epidermidis* and *E. coli*. solution co-cultured with the hydrogels after 1 day. **b** The bactericidal ratio of the hydrogels to *S. epidermidis* and *E. coli*. (Error bar means the standard deviation, * indicates statistically difference at *p* < 0.05, *p* value was generated by one‐way analysis of variance (ANOVA), followed by Tukey's multiple‐comparison post hoc test, *n* = 3). **c** Scheme of the in vivo antibacterial experiments. **d** Photographs of harvested hydrogels after they were implanted in the skin pockets for 7 days of post surgery. **e** Hematoxylin–eosin (H&E)-stained sections of connective tissues surrounding the hydrogel

The NP-P-PAA hydrogels displayed strong antibacterial activities owing to the broad-spectrum antimicrobial activity of Ag in the Ag-Lignin NPs^46, 47^. Figure 4a shows a photograph of bacterial suspensions cultured with hydrogels. The suspensions of the blank group, PAA, and P-PAA were turbid during the 24-h culture, whereas that of the NPs-P-PAA hydrogel was clear. The bactericidal ratio of the hydrogel for *Escherichia* *coli* and *Staphylococcus epidermidis* were 97% and 98%, respectively (Fig. 4b). These results indicated the hydrogel effectively and significantly inhibited both *G−* and *G*+ bacteria.

The antibacterial activities of the hydrogel were further confirmed in vivo in a rabbit model. The purified NPs-P-PAA hydrogels were implanted in the subcutaneous pockets on the back of rabbits and then an *E. coli* suspension (1 mL, 10^5^ cells mL^−1^) was injected in the pockets (Fig. 4c). After 7 days, the surgical sites were harvested to examine the infections and inflammatory reactions (Fig. 4d). The site treated with P-PAA was filled with purulence, whereas the site treated with NPs-P-PAA was clear. The tissue surrounding the implants was removed and stained with hematoxylin and eosin (H&E) to assess the anti-infection ability of the hydrogel (Fig. 4e). Histological staining revealed that the P-PAA group had a large number of multinucleated giant cells and edema tissue in the surrounding tissue, and the subcutaneous tissue was destroyed, indicating that the *E. coli* caused severe inflammation reactions. In sharp contrast, the tissue surrounding the NPs-P-PAA hydrogel was in good condition and there were almost no multinucleated giant cells or edema tissue around the hydrogel. These results demonstrated that the NPs-P-PAA hydrogel had good antibacterial ability in vivo and can be safely applied for healing wounds and repairing bone or cartilage.

### Cell affinity and wound healing

**Fig. 5:**
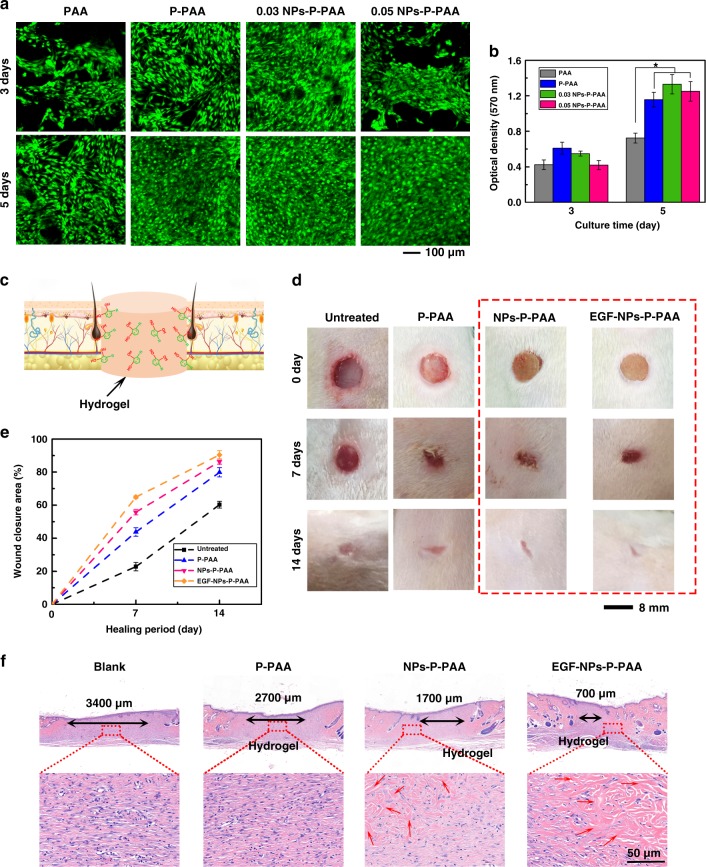
The biocompatible NPs-P-PAA hydrogel used to repair a full-thickness skin defect. **a** Confocal laser scanning microscopy (CLSM) micrographs of fibroblasts on various hydrogels. **b** MTT assay of the proliferation of fibroblasts. (Error bar means the standard deviation, * indicates statistical difference at *p* < 0.05, *p* value was generated by one‐way analysis of variance (ANOVA), followed by Tukey's multiple‐comparison post hoc test, *n* = 4). **c** Scheme of the hydrogel implanted into the skin defect of a rat. **d** Percent wound closure at different periods of post wounding. **e** Representative of the gross appearance of defects treated with various hydrogels. **f** Hematoxylin–eosin (H&E) staining of the wound section after 14 days of treatment. NPs, nanoparticles; P, pectin; PAA, polyacrylic acids

The NPs-P-PAA hydrogel exhibited cell affinity and favored the adhesion and proliferation of cells (Fig. 5a, b). Fibroblasts, the cell types typically responsible for wound repair, were cultured on the PAA, P-PAA, and NPs-P-PAA hydrogels. Before cell culture or the implantation experiment, the hydrogel was purified by repeated purification in a phosphate-buffered saline solution and 75% alcohol to remove excessive APS and other residues. Confocal laser scanning microscopy images showed that all the hydrogels supported cell adhesion and spreading (Fig. 5a). Compared with the PAA hydrogel, the P-PAA hydrogel showed better cell adhesiveness. The 0.03 NPs-P-PAA hydrogel showed the best cell adhesiveness. An MTT (3-[4,5-dimethylthiazol-2-yl]-2,5-diphenyl tetrazolium bromide) assay was used to further evaluate the cell proliferation on the hydrogel (Fig. 5b). The proliferation of fibroblasts on the P-PAA and NPs-P-PAA hydrogels was faster than that on the PAA hydrogel because the pectin and Ag-Lignin NPs had improved biocompatibility and cell affinity. Note that the silver release of the current hydrogel was lower than that in an earlier study^48^ (Supplementary Figure 23 and Supplementary Notes 22), which is because both the lignin and the hydrogel system play important roles in retarding the release of Ag^+^. The functional groups of lignin can bind Ag^+^ to slow Ag release^49, 50^. In addition, the functional groups of PAA^51, 52^ and pectin^53^, such as carboxyl and hydroxyl, coordinate with Ag^+^. Thus, the concentrations of released Ag^+^ are low and such a low dose of Ag^+^ is non-toxic to cells.

The NPs-P-PAA hydrogel was further used to repair full-thickness skin defects in vivo (Fig. 5c–f). The NPs-P-PAA hydrogel showed better healing than the blank and P-PAA. After epidermal growth factor (EGF) was loaded, the hydrogels showed the best healing performance. After implantation of the hydrogel for 14 days, the wound was well healed and the defect areas were nearly closed in all the groups treated with a hydrogel, whereas large scars were observed only in the groups treated without hydrogel (Fig. 5d). The skin healing ratio, which was defined as the ratio of wound healing area to the initial defect area, was used to quantitatively evaluate the wound healing rate of different hydrogel-treated defect areas (Fig. 5e). The NPs-P-PAA hydrogel had a healing ratio of 90%, which was higher than that of the blank group (59%) and the P-PAA hydrogel (78%). The EGF-loaded hydrogel had the highest healing ratio of 92%.

The quality of the regenerated skin tissue in the defects was further investigated by H&E staining. As shown in Fig. 5f, all the samples were covered with an intact and complete layer of epidermis. The samples treated with a blank and P-PAA had a large area of unmatured tissue, whereas the sample treated with NPs-P-PAA hydrogel had a small proportion of new tissue. The quality of the newly regenerated tissue was further examined by obtaining magnified micrographs. Many granulation tissue still existed in the regenerated area of the samples treated with the blank and P-PAA hydrogel, whereas collagen fibers appeared in the sample treated with NPs-P-PAA hydrogels. The sample treated with EGF-loaded hydrogel had ordered collagen fibers and hair follicles, which showed that this regenerated tissue was almost mature. In short, this NPs-P-PAA hydrogel was able to repair wound healing and increase skin tissue regeneration, and it had better tissue regeneration ability than the other hydrogels.

## Discussion

In summary, we prepared a plant catechol-chemistry-based hydrogel with high adhesiveness, toughness, and cell affinity. Ag-Lignin NPs were developed to generate radicals and maintain the quinone-catechol redox balance in the inner hydrogel network. Compared with the commonly used adhesives, this hydrogel had the following advantages. First, the gelation of the hydrogel was triggered by radically enriched Ag-Lignin NPs without the need for UV and thermal treatment. Therefore, the hydrogel was biocompatible and not harmful to skin tissue. Second, the hydrogels displayed durable adhesiveness and maintained their adhesiveness for a long time. Third, the plant-inspired Ag-Lignin NPs hydrogel exhibited good cell affinity and tissue adhesiveness. Finally, the hydrogel has the ability of anti-infection, which is particularly suitable for skin wound repair. In short, this easy-to-prepare and environmentally friendly plant-inspired hydrogel illustrates a strategy for the development of adhesiveness hydrogels with multifunctionality based on dynamic redox catechol chemistry.

## Methods

### Materials

Alkali lignin (Wn = 1000~10,000) was purchased from Qunlin paper Group Co., China, pectin (P, galacturonic acid content ≥74%) and AA (99.0%) were supplied by Macklin, APS (98.0%) and poly (ethylene glycol) dimethacrylate (PEGDA, average Mn = 750, 99.8%) were purchased from Sigma-Aldrich (USA). AgNO_3_ (99.8%), ammonia solution (NH_3_·H_2_O, 25.0∼28.0%), and sodium hydroxide (NaOH, 99.0%) was purchased from KESHI Chemical Works in Chengdu. Deionized water was used in the experiment. All solvents and chemicals were purchased from commercial sources and used as received, unless otherwise noted.

### Preparation of Ag-Lignin NPs

The Ag-Lignin core-shell NPs were prepared according to the procedure described in previous report^50^. First, an aqueous solution of lignin at a concentration of 50 mg mL^−1^ was prepared by dissolving the weighted amount of lignin powder in NaOH solution (pH = 10) with the aid of ultrasonic agitation (solution A). Second, an aqueous solution of AgNO_3_ at an Ag^+^ ion concentration of 3.33, 10, and 16.67 mg mL^−1^ was prepared and 5 mol L^−1^ of aqueous ammonia solution was added to the silver-ammonia complex (solutions B). Finally, solution A was added slowly dropwise to solution B and reacted at room temperature for 1 h to obtain Ag-Lignin core-shell NPs (Ag-Lignin NPs) solution. The concentration of various Ag-Lignin NPs was listed in Supplementary Notes 1 and Supplementary Table 1. CV was used to analyze the reaction between lignin and [Ag(NH_3_)_2_]^+^ solution (Supplementary Notes 7).

### Preparation of hydrogel

AA, pectin, Ag-Lignin NPs solution, APS, PEGDA, and ceionized water were poured into the beaker and stirred to prepare a homogeneous solution. Thereafter, the solution was injected into a reaction mold. Finally, the samples were placed in N_2_ atmosphere for 20 min at room temperature to obtain NPs-P-PAA hydrogels. The formulations of hydrogels were denoted as *x* NPs-P-PAA, where *x* was masses of AgNO_3_. The details of preparation and composition of various hydrogels were listed in Supplementary Notes 11, 12 and Supplementary Table 5.

### Characterization

The electron spin-resonance spectroscopy analysis results were performed on an ESR Spectrometer (JES-FA200 ESR Spectrometer, Japan) at 9.873 GHz. The morphology structures were examined using a scanning electron microscope (SEM; JSM 6390, JEOL, Japan). TEM images of NPs in aqueous dispersion were obtained by using a Tecnai-F30, FEI, USA. The mechanical property measurements and adhesiveness properties of the hydrogels were conducted according to our previous studies^25, 54^ using a universal testing machine (5567, Instron, America) with a 100 N load cell. Details are described in Supplementary Notes 2–6 and 17–20.

### Antibacterial activity in vitro

To investigate the antibacterial activity of the hydrogel, *S. epidermidis* (ATCC6538, Gram-positive organism) and *E. coli* (ATCC8739, Gram-negative organism) were used for the tests according to our previous study^54^. The antibacterial activity of five groups of samples (30 μg sample^−1^), including PAA, P-PAA, and NPs-P-PAA with different concentrations of NPs, were tested by evaluating the inhibition of the bacterium *S. epidermidis* and *E. coli*. Details are described in Supplementary Notes 23.

### Antibacterial activity in vivo

The antibacterial activities of the hydrogel were further confirmed in vivo in a rabbit model, refer to our previous study^54^. The P-PAA and NPs-P-PAA hydrogels were implanted subcutaneously on the back of New Zealand rabbit (Dashuo, Chengdu, China), following which 1 mL of *E. coli* (10^5^ cells mL^−1^) was injected to the hydrogel site. The P-PAA hydrogels were treated as a positive control. In the days following the surgical operation, the animal activity and appearance of the wound was examined each day. The surgical sites were harvested to examine infections and inflammatory reactions (Supplementary Notes 24).

### Cell biocompatibility in vitro

NIH-3T3 fibroblast (SCSP-515, Stem Cell Bank, Chinese Academy of Sciences, Shanghai, China) cells in a growth phase were treated with trypsin and harvested. According to previous methods^54^, the cells were seeded on the hydrogels with a density of 5 × 10^4^ cells) in the wells of the tissue culture plates and left undisturbed in an incubator for 3 h to allow for cell attachment. Then, an additional 1 mL of the Dulbecco’s modified eagle medium (HyClone, USA) supplemented with 10% fetal bovine serum (HyClone, USA) was added into each well. The cells were allowed to adhere and grow for 3 and 5 days. The morphologies of the cells on the hydrogel surfaces were observed using a laser scanning confocal microscope (Leica, Germany). The biocompatibility of the hydrogel and the cell proliferation was assessed by the MTT assay. Details were described in Supplementary Notes 25.

### Wound healing in vivo

Full skin wounds were created on the dorsal area of rats and treated with the P-PAA hydrogel, NPs-P-PAA hydrogels, and the EGF-loaded NPs-P-PAA hydrogels. The wounds treated without hydrogels were used as a control. The surgical procedure was performed according to a previous study^55^. Briefly, four full-thickness circular wounds (8 mm in diameter) were created on the upper back of each Sprague Dawley (180∼220 g, Dashuo, Chengdu, China) rat using a disposable 8-mm skin biopsy punch. The P-PAA, NPs-P-PAA, and EGF-loaded hydrogels (30 μg sample^−1^, Shanghai Primegene Bio-Tech Co., Ltd.) were implanted on other wound sites of the rats. A wound without a hydrogel was used as a control. Five parallel specimens of each type of hydrogel were tested. All animal procedures were performed according to protocols approved by the institutional animal ethics committee of the Southwest Jiaotong University and laboratory animal administration rules of China. Details were described in Supplementary Notes 26.

## Supplementary information

Supplementary Information

## Data Availability

The authors declare that all relevant data of this study are available from the corresponding authors.
