# Supplementary material for: Plant-inspired adhesive and tough hydrogel based on Ag-Lignin nanoparticles-triggered dynamic redox catechol chemistry
Source: Nat Commun. 2019 Apr 2;10:1487. doi: 10.1038/s41467-019-09351-2 (PMC6445137; doi:10.1038/s41467-019-09351-2)
Supplement: Supplementary file 1 — Supplementary Information [file 41467_2019_9351_MOESM1_ESM.pdf]

Supplementary Information

Plant-Inspired Adhesive and Tough Hydrogel Based on Ag-Lignin Nanoparticles Triggered Dynamic Redox Catechol Chemistry

*Donglin Gan, Wensi Xing, Lili Jiang, Ju Fang, Cancan Zhao, Fuzeng Ren, Liming Fang, Kefeng Wang, and Xiong Lu**

Supplementary Figures

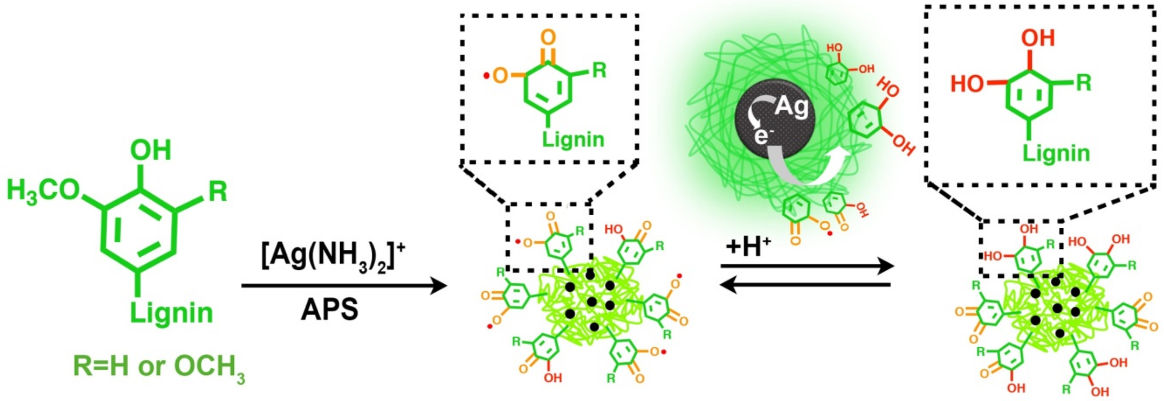

Supplementary Figure 1 Preparation mechanism of Ag-Lignin NPs

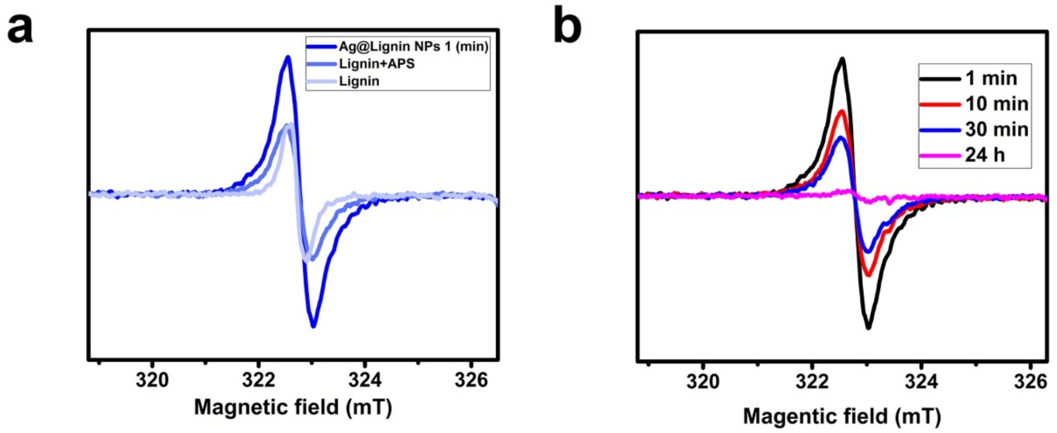

Supplementary Figure 2 a) ESR spectra of the fresh solutions (Ag-Lignin NPs, lignin, lignin and APS) prepared after 1 min and b) ESR spectra of the Ag-Lignin NP solutions prepared after 1 min, 10 min, 30 min, and 24 h

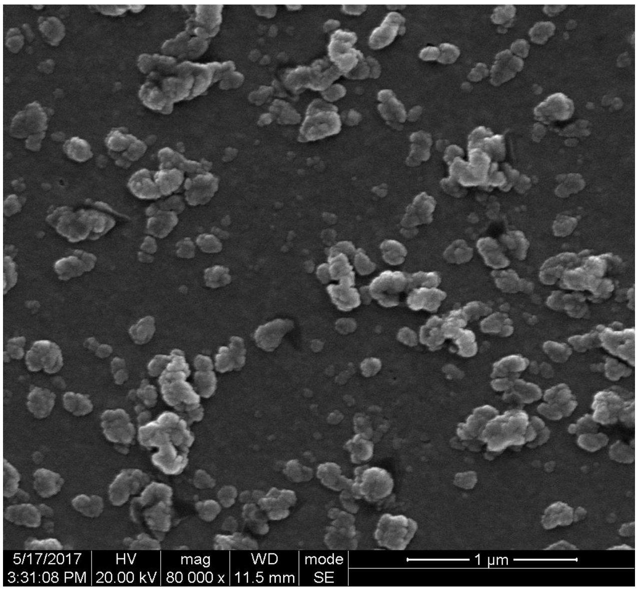

Supplementary Figure 3 SEM of Ag-Lignin NPs

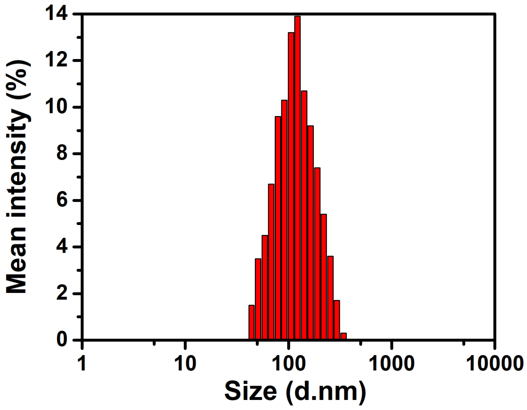

Supplementary Figure 4 DLS analysis of the Ag-Lignin NPs

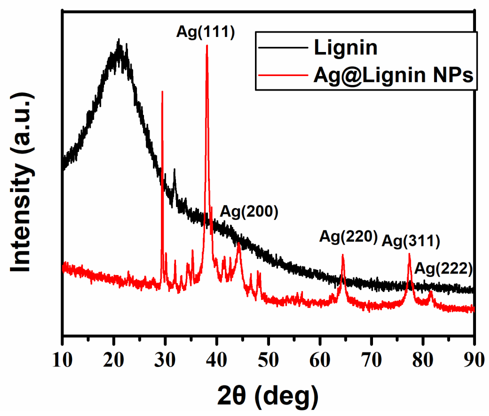

Supplementary Figure 5 XRD patterns of the Ag-Lignin NPs and lignin

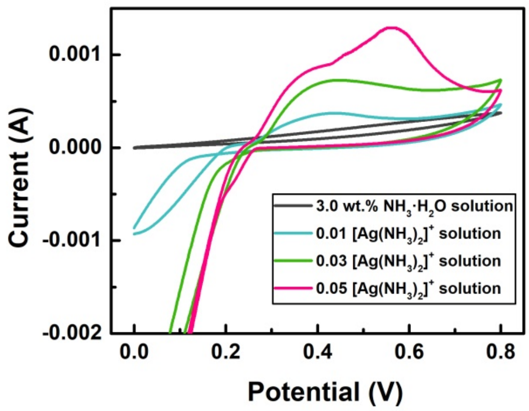

Supplementary Figure 6 CV of the lignin working electrode in $NH_3·H_2O$ and $[Ag(NH_3)_2]^+$ solutions with different concentration. The scan speed was 5 mV s^{-1}.

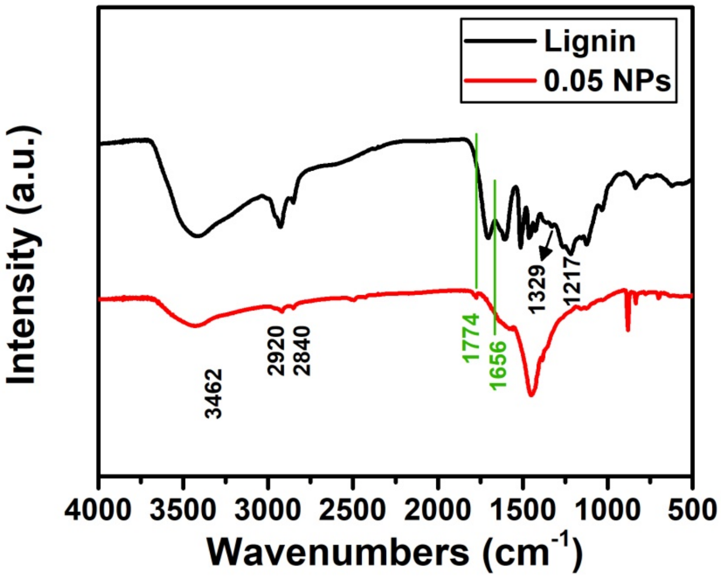

Supplementary Figure 7 FT-IR spectra of the Ag-Lignin NPs and lignin

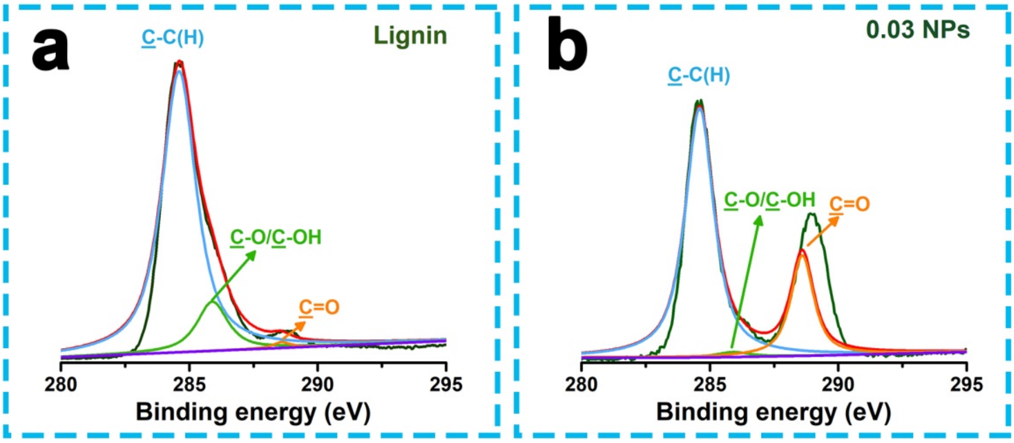

Supplementary Figure 8 XPS spectra in the C1s regions of (a) lignin, (b) lignin after oxidation for 24 h by $[Ag(NH_3)_2]^+$.

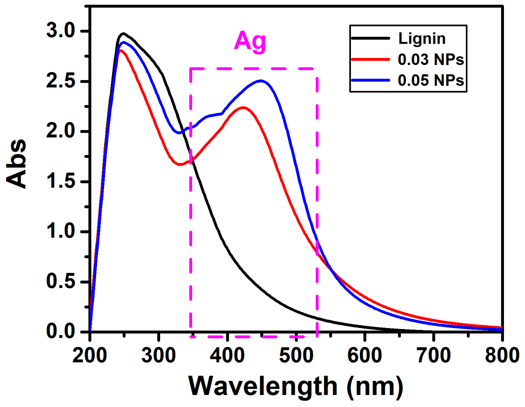

Supplementary Figure 9 UV-vis of Ag-Lignin NPs and lignin

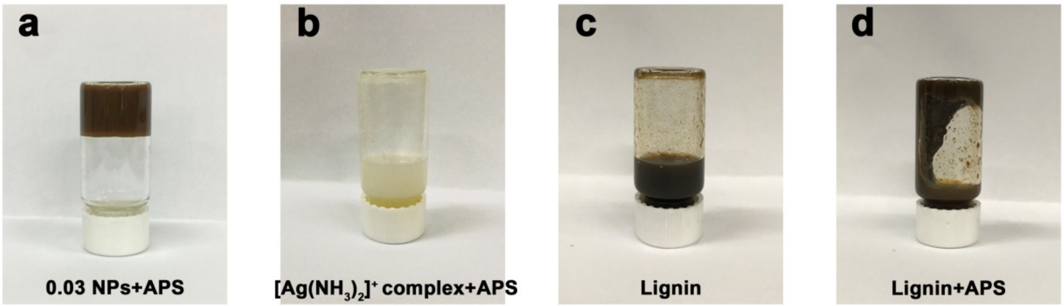

Supplementary Figure 10 Gelation of the various P-PAA hydrogels triggered by different systems. (a) by 0.05 Ag-Lignin NPs, which cause gelation. (b) by the $[Ag(NH_3)_2]^+$ complex, which does not cause gelation. (c) by lignin, which does not cause gelation. (d) by lignin and APS, which does not cause gelation. These images prove that Ag-Lignin NPs are the main trigger of gelation.

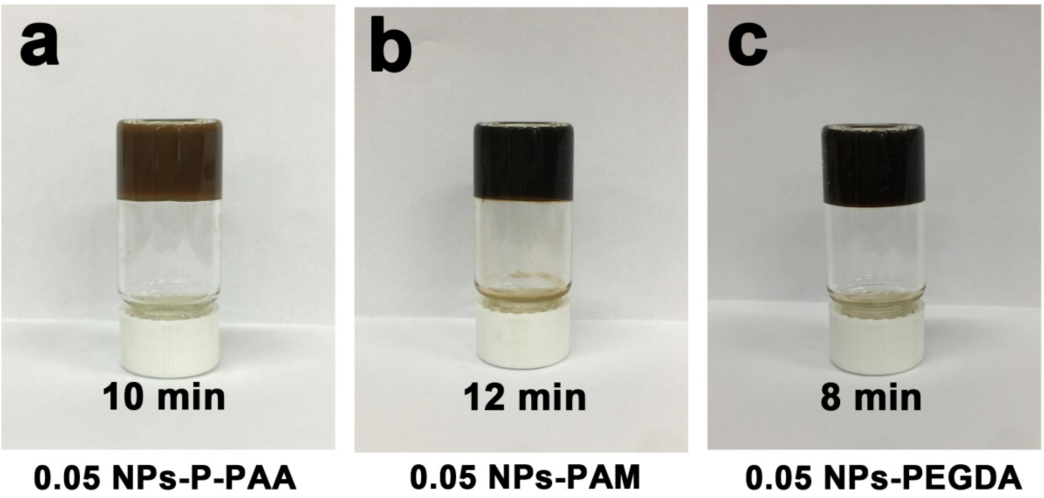

Supplementary Figure 11 Gelation of the various hydrogels triggered by Ag-Lignin NPs. (a) 0.05 NPs-P-PAA. (b) 0.05 NPs-PAM. (c) 0.05 NPs-PEGDA.

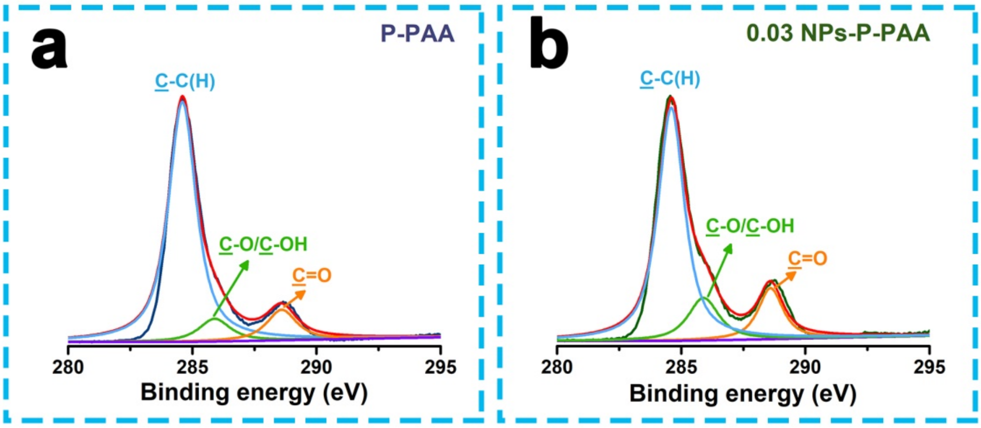

Supplementary Figure 12 XPS analysis on the redox reaction of the hydrogel. (a) XPS spectra in the C1s regions of the P-PAA hydrogel and (b) 0.03 NPs.

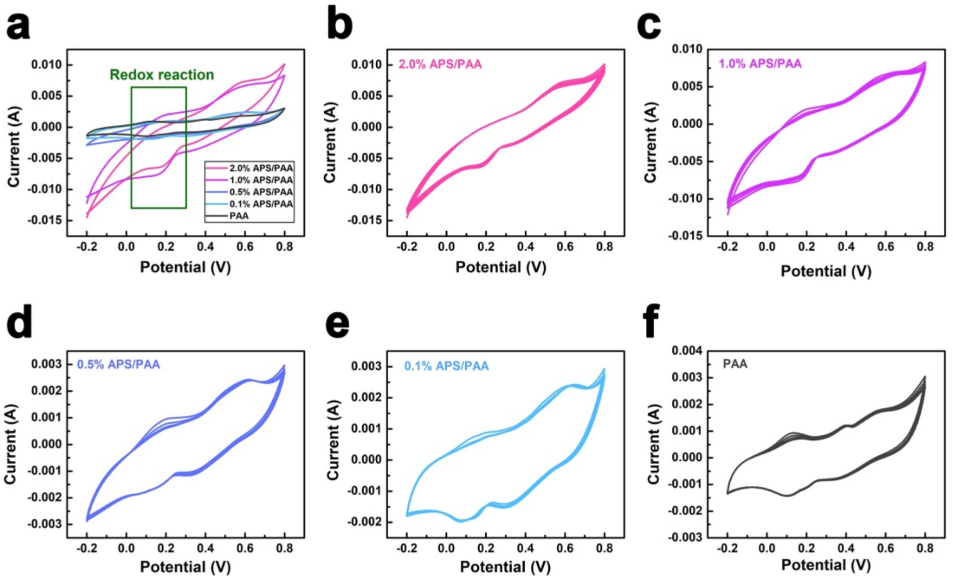

Supplementary Figure 13 CV experiment to analyze the dynamic redox reaction of the hydrogel. CV of the NPs working electrodes in 10% PAA solution with different contents of ammonium persulfate (APS) over (a) one cycle and (b-f) four cycles. The scan speed was 5 mV s^{-1}.

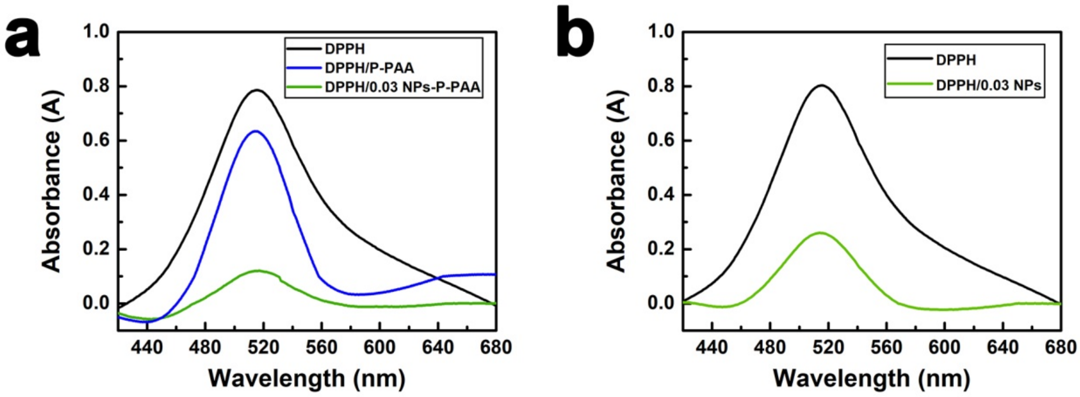

Supplementary Figure 14 UV-vis spectra of DPPH and scavenging by (a) 0.03 NPs-P-PAA and P-PAA hydrogels and (b) 0.03 NPs for 30 min.

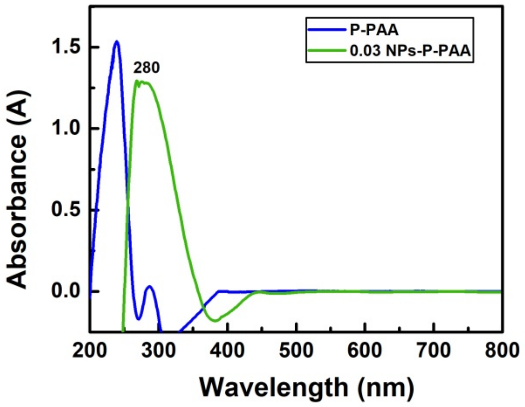

Supplementary Figure 15 UV-vis spectra of P-PAA and the 0.03 NPs-P-PAA polymeric system without crosslinking

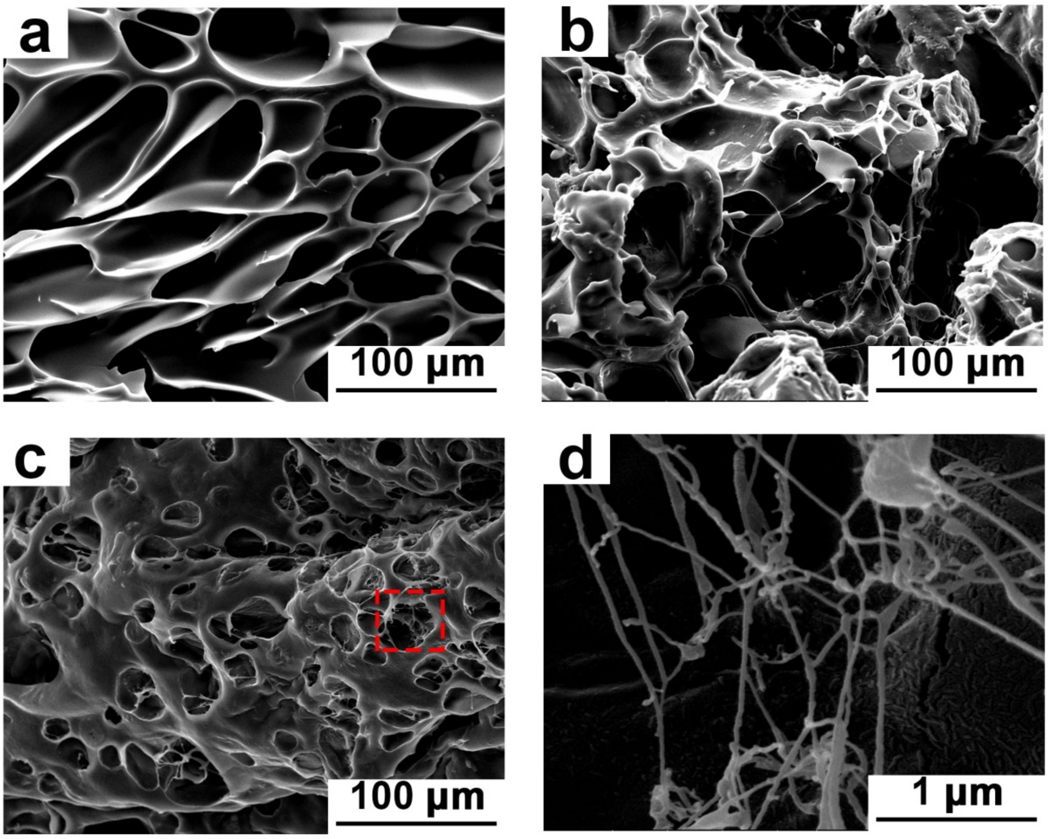

Supplementary Figure 16 SEM images of a) the PAA hydrogel, b) P-PAA hydrogel, c) 0.05 NPs-P-PAA hydrogel, and d) microfibrils embedded in the 0.05 NPs-P-PAA hydrogel

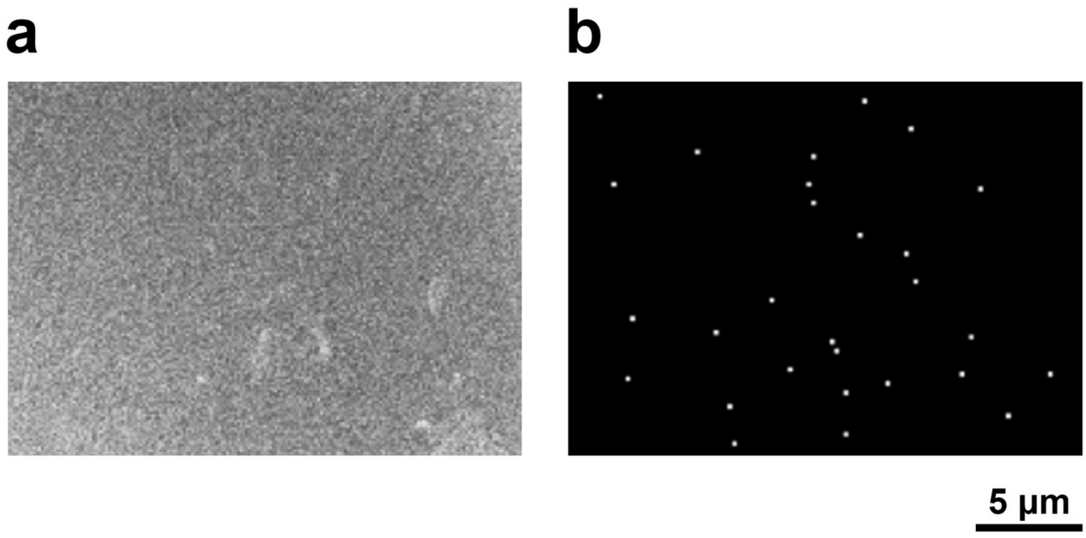

Supplementary Figure 17 a) SEM micrograph of the 0.03 NPs-P-PAA hydrogel. b) Element mapping of Ag in the hydrogel shown in (a).

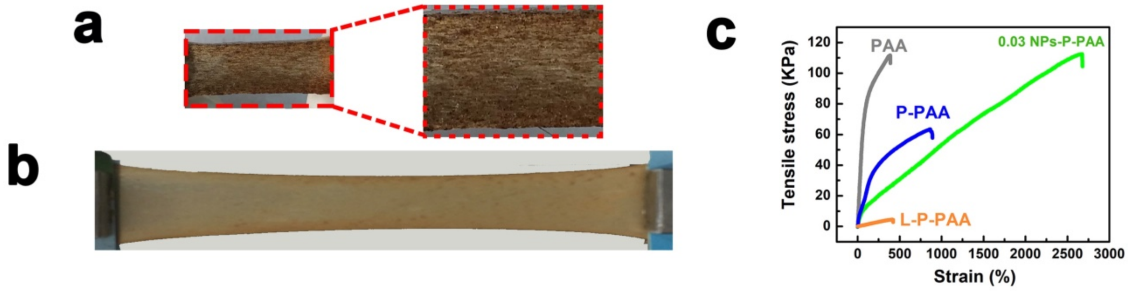

Supplementary Figure 18 Tensile test of the hydrogel with pure lignin. (a) Photos of L-P-PAA hydrogel, (b) Photos of 0.05 NPs-PAA hydrogel after tensile tests. (c) Typical tensile stress-strain curves of the hydrogels.

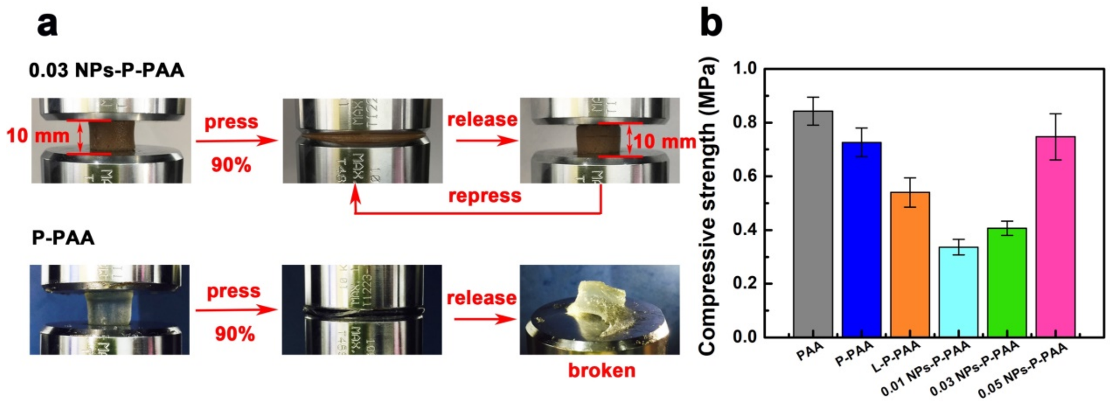

Supplementary Figure 19 a) The 0.03 NPs-P-PAA hydrogel recoved within 2 min after compression, and the PAA hydrogel was broken after compression. b) The compressive strengths of various hydrogels. (Error bar means the standard deviation, n=4)

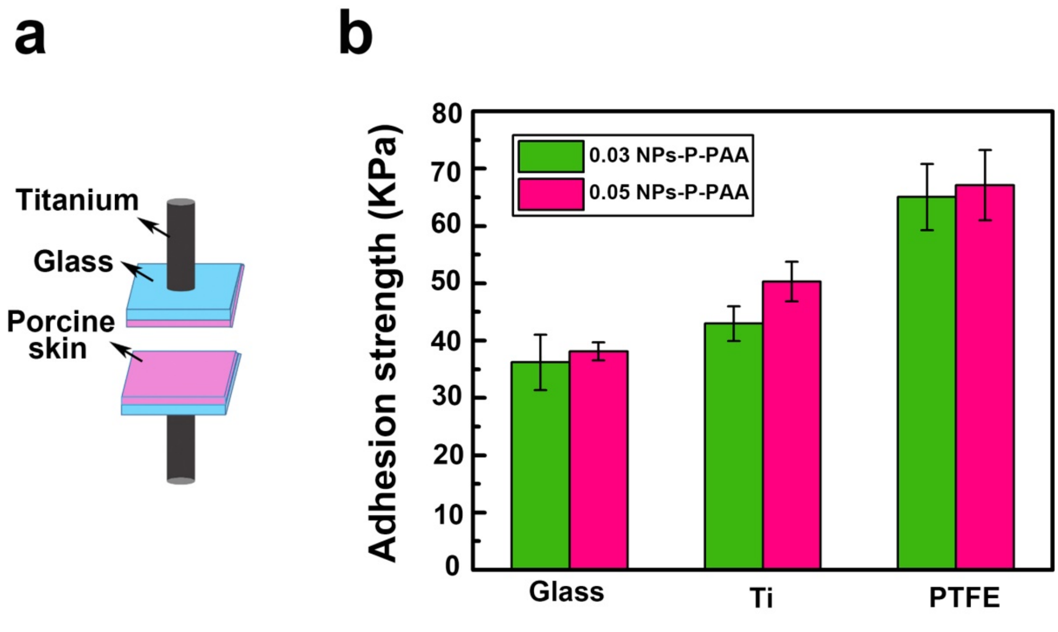

Supplementary Figure 20 (a) Schematics of tensile-adhesion testing, (b) The adhesive strength of the hydrogels to various material surfaces. (Error bar means the standard deviation, n=4)

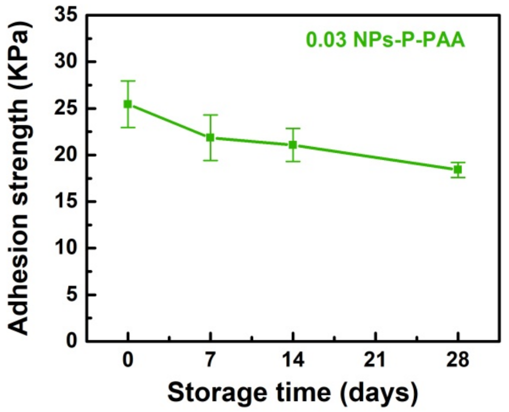

Supplementary Figure 21 Long-term adhesiveness of the 0.03 NPs-P-PAA hydrogel after 7, 14, and 28 days of storage. (Error bar means the standard deviation, n=4)

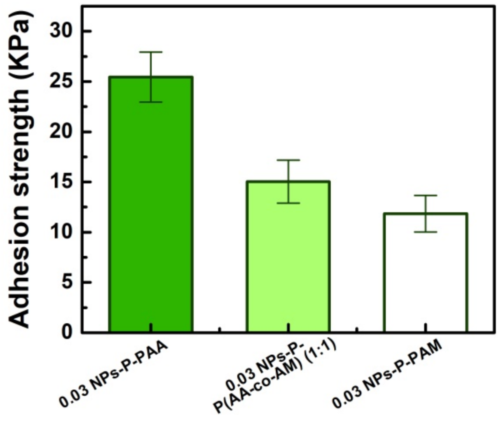

Supplementary Figure 22 Adhesion strengths of 0.03 NPs-P-PAA, 0.03 NPs-P-P(AA-co-AM), and 0.03 NPs-P-PAM hydrogels. (Error bar means the standard deviation, n=4)

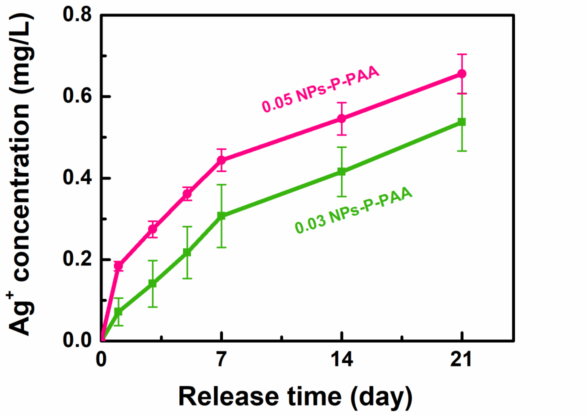

Supplementary Figure 23 In vitro release profiles of Ag^+ from the various NPs-P-PAA hydrogels. (Error bar means the standard deviation, n=4)

Supplementary Tables

Supplementary Table 1 Content of the different Ag-Lignin NPs

Ag-Lignin NPs	$AgNO_3$ (g)	NaOH (g)	Lignin (g)	Deionized Water (mL)
0.01 NPs	0.01	0.05	0.05	4
0.03 NPs	0.03	0.05	0.05	4
0.05 NPs	0.05	0.05	0.05	4

*Ag-Lignin NPs are denoted as x NPs, where x is the mass of $AgNO_3$.

Supplementary Table 2 Content of the various solutions for the ESR analysis

Samples	0.05 NPs	Lignin	APS
0.05 NPs	0.0250 g mL^{-1}	/	/
0.05 NPs and APS	0.0250 g mL^{-1}	/	0.0250 g mL^{-1}
Lignin	/	0.0125 g mL^{-1}	/
Lignin and APS	/	0.0125 g mL^{-1}	0.0250 g mL^{-1}

Supplementary Table 3 Peak areas and g-values of the ESR spectra

Samples	g-value	IH
NPs-APS	2.00340	29.41
Lignin-APS	2.00335	5.07

Supplementary Table 4 The compositions of the $NH_3 \cdot H_2O$ and $[Ag(NH_3)_2]^+$ solutions

Solution	$AgNO_3$ (g)	$NH_3 \cdot H_2O$ (mL)	Deionized Water (mL)
3.0 wt. % $NH_3 \cdot H_2O$ solution	/	2.5	200
0.01 $[Ag(NH_3)_2]^+$ solution	0.01		
0.03 $[Ag(NH_3)_2]^+$ solution	0.03	5 mol L^{-1} aqueous ammonia solution	
0.05 $[Ag(NH_3)_2]^+$ solution	0.05		

Supplementary Table 5 Content of the various hydrogels

Hydrogels	AA (mL)	Pectin/ AA (wt.%)	APS/ AA (wt.%)	PEGDA/ AA (wt.%)	NPs (mL)	Deionized Water (mL)	Water content (wt. %)
PAA Hydrogels	2.7	0	5	0.1	0	10	79
P-PAA Hydrogels	2.7	10	5	0.1	0	10	77
0.01 NPs-P-PAA Hydrogels	2.7	10	5	0.1	4 （0.01 NPs）	6	77
0.03 NPs-P-PAA Hydrogels	2.7	10	5	0.1	4 （0.03 NPs）	6	77
0.05 NPs-P-PAA Hydrogels	2.7	10	5	0.1	4 （0.05 NPs）	6	77

Supplementary Table 6 Peak species, binding energies, and peak area ratios (%) of the high-resolution C1s spectra of lignin, the 0.03 NPs, and the 0.03 NPs hydrogel

Peak species	C-C(H)	C-O(C-OH)	C=O	C-O(C-OH) / C=O ratio
Binding energy (eV)	284.6	285.9	288.6	
Lignin	85.61%	13.49%	0.90%	15.01
0.03 NPs	74.56%	1.67%	23.78%	0.07
P-PAA hydrogel	80.47%	8.60%	10.94%	0.79
0.03 NPs-P-PAA hydrogel	70.61%	15.04%	14.35%	1.05

The C-O/C=O ratios were calculated according to the peak areas of C-O and C=O from the XPS spectra.

Supplementary Table 7 The compositions of different APS-PAA solutions

Solution	APS (g)	PAA (g)	Deionized Water (mL)
PAA	/	10	100
0.1% APS-PAA	0.1	10	100
0.5% APS-PAA	0.5	10	100
1.0% APS-PAA	1.0	10	100
2.0% APS-PAA	2.0	10	100

Supplementary Table 8 Compositions of the DPPH, DPPH/0.03 NPs, DPPH/P-PAA, and DPPH/0.03 NPs-P-PAA solutions

Solution	DPPH (mg)	Reacted materials (freeze-dried)	Methanol (mL)
DPPH	0.4	/	10
DPPH/0.03 NPs	0.4	50 mg 0.03 NPs	10
DPPH/P-PAA	0.4	50 mg P-PAA	10
DPPH/0.03 NPs-P-PAA	0.4	50 mg 0.03 NPs-P-PAA	10

Supplementary Table 9 The compositions of 0.03 NPs hydrogels with different monomers

Hydrogel	0.03 NPs (mL)	Pectin (g)	AA (g)	AM (g)
0.03 NPs-P-PAA	4	0.27	2.7	0
0.03 NPs-P-P(AA-co-AM)	4	0.27	1.35	1.35
0.03 NPs-P-PAM	4	0.27	0	2.7

Supplementary Notes

Supplementary Notes 1: Preparation of Ag-Lignin NPs

Ag-Lignin core-shell NPs were prepared according to the procedure described in a previous report[1]. First, an aqueous solution of lignin at a concentration of 50 mg mL^{-1} was prepared by dissolving the weighed amount of lignin powder in an NaOH solution (pH = 10) with the aid of ultrasonic agitation (solution A). Second, aqueous solutions of $AgNO_3$ with an Ag^+ ion concentration of 3.33, 10, and 16.67 mg mL^{-1} were prepared, and the aqueous ammonia solution at 5 mol L^{-1} was added to the silver-ammonia complex (solutions B). Finally, solution A was added dropwise to solution B and reacted at room temperature for 1 h to obtain an Ag-Lignin core-shell NP solution. The concentrations of the various Ag-Lignin NPs are listed in Supplementary Table 1.

Supplementary Notes 2: Electron spin resonance spectroscopy (ESR) analysis

ESR analysis was performed on an ESR Spectrometer (JES-FA200 ESR Spectrometer, Japan) at 9.873 GHz. To ascertain the free radicals in this system, the mixture solutions of 0.05 NPs, 0.05 NPs with APS, lignin, and lignin with APS were tested. The concentrations of the various solutions for the ESR analysis are listed in Supplementary Table 2. The solutions were rapidly transferred to a standard capillary and placed in the EPR spectrometer. The spectrum was recorded. To determine the type of radicals, the g-values were calculated according to the following equation[2].

$$g = \frac{hv}{\mu_B B} = 0.07144773 \frac{v}{B} \qquad \textbf{(1)}$$

The parameter h is the Planck constant, and v is the frequency of the electromagnetic radiation. The parameter B is the magnetic field, and μ_B is the Bohr magneton.

ESR analysis revealed that the lignin solution contained quinone (semiquinone) radicals. The addition of APS into the lignin solution did not change the number of radicals in the solution (Supplementary Figure 1). However, the number of radicals increased after $[Ag(NH_3)_2]^+$ was mixed with the lignin, which was more than that of the lignin and lignin with APS. After the 24 h reaction, Ag-Lignin was formed and mixed with APS. The numbers of radicals increased to five times that of the lignin solution (Supplementary Table 3).

Based on the ESR analysis (Supplementary Figure 2), the mechanism of the free radical generation was determined as follows. First, the hydroxyl and methoxy functional groups on the aromatic rings of the lignin were oxidized by $[Ag(NH_3)_2]^+$ to become quinone (semiquinone) radicals. Concurrently, the Ag-Lignin NPs were formed. After APS was added to the Ag-Lignin NP solution, the quinone (semiquinone) groups were activated and formed more radicals to initiate AA polymerization. Conversely, the quinone radicals (semiquinone radicals) on the Ag-Lignin NPs were converted to catechol groups dynamically in the hydrogel system, which produced hydrogels with excellent and long-lasting adhesion.

Supplementary Notes 3: Scanning electron microscope (SEM) morphology

The morphologies of the Ag-Lignin NPs were examined using a scanning electron microscope (SEM; JSM 6390, JEOL, Japan). Before examination, the Ag-Lignin NPs solutions were freeze-dried. Then, the dried Ag-Lignin NPs were exposed under SEM (Supplementary Figure 3).

Supplementary Notes 4: Dynamic Light Scattering (DLS) analysis

DLS (ZETA-AIZER, Malvern, UK) analysis showed a diameter of 135 ± 10 nm (Supplementary Figure 4). Before examination, the Ag-Lignin NPs solutions were diluted in

deionized water.

Supplementary Notes 5: High-resolution transmission electron microscopy (HRTEM)

TEM images of the nanoparticles in the aqueous dispersion were obtained using a Tecnai-F30, FEI, USA. The samples were prepared by dropping the prepared aqueous dispersion onto amorphous carbon-coated copper grids and dried.

Supplementary Notes 6: X-ray diffraction (XRD) analysis of the lignin and Ag-Lignin NPs

The XRD patterns of the Ag-Lignin NPs and lignin were obtained using an X-ray diffractometer (X'pert PRO, Philips, The Netherlands) at a voltage of 40 kV and a current of 40 mA. Cu Kα filtered radiation (λ = 1.5406 nm) was used. Before examination, the Ag-Lignin NPs solutions were freeze-dried to obtain dried Ag-Lignin NPs.

Supplementary Notes 7: Cyclic voltammetry (CV) analysis of lignin reacting with $[Ag(NH_3)_2]^+$ solution

Previous studies have proven that a redox reaction occurs between $[Ag(NH_3)_2]^+$ and lignin[3, 4]. Cyclic voltammetry (CV) was processed to characterize the reaction between $[Ag(NH_3)_2]^+$ and lignin, and found distinct redox peaks in the spectra. The CV was conducted using an Electrochemical Workstation (IM6 Instruments, Zahner, Germany). Lignin was coated on nickel foam and used as the working electrode; Ag/AgCl (KCl sat.) was the reference electrode; Pt was the counter electrode. Aqueous solutions of $[Ag(NH_3)_2]^+$ with different concentrations were used as the electrolyte (1 M) (Supplementary Table 4). A pure $NH_3{\cdot}H_2O$ solution was used as the control electrolyte. The scan rate of CV was set at 5 mV s^{-1} with the potential range from -0.20 to 0.80 V versus Ag/AgCl.

The results are shown in Supplementary Figure 6. Reversible oxidation peaks at 0.32 V and 0.58 V appeared in the $[Ag(NH_3)_2]^+$ electrolyte, which corresponded to quinone/catechol conversion in lignin caused by $[Ag(NH_3)_2]^+$ oxidation. In contrast, redox couple peaks did not appear in the $NH_3 \cdot H_2O$ electrolyte. These results prove that the oxidation of lignin occurs during the CV process, in which the methoxyl groups and catechol groups of lignin are oxidized into quinone groups by $[Ag(NH_3)_2]^+$.

Supplementary Notes 8: FT-IR analysis of the lignin and Ag-Lignin NPs

To further reveal the oxidation mechanism, Fourier transform-infrared spectroscopy (FT-IR; Nicolet 5700, Thermo, USA) analysis was performed on the lignin and Ag-Lignin NPs (Supplementary Figure 7). In the FT-IR spectrum of lignin, a sharp peak at 2920 cm^{-1} was assigned to methyl and methylene groups. A symmetric stretch for $-CH_3$ in the methoxyl groups appeared at 2840 cm^{-1}. The peak at 1217 cm^{-1} was assigned to syringyl ring breathing with C-O stretching. In the FT-IR spectrum of the Ag-Lignin NPs, these peaks decreased in intensity or disappeared, which proved that the methoxy groups were removed during the process of lignin oxidation by silver. The intensity of bands at 3462 cm^{-1} and 1329 cm^{-1}, representing the O-H stretching vibrations, decreased significantly after lignin was impregnated in the $[Ag(NH_3)_2]^+$ solution. In addition, bands appeared at 1656 and 1774 cm^{-1} for the C=O stretching vibration. These results prove that the catechol groups and methoxy groups of lignin are oxidized to quinone groups by Ag^+ ions.

Supplementary Notes 9: XPS analysis of the lignin and Ag-Lignin NPs

The chemical compositions of lignin and the 0.03 NPs were measured using XPS (Kratos, Axis Ultra DLD, Manchester, UK). A monochromatic Al Kα X-ray excitation source (hv

=1486.6 eV) was used at 15 kV and 150 W. The C1s spectra at the binding energy of 285 eV was set as a reference. The samples were freeze-dried at -80 °C before analysis.

The XPS results indicated that lignin had high contents of C-O and C-OH groups 285.9 eV and a low content of C=O at 288.6 eV (Supplementary Figure 8). For the Ag-Lignin NPs, the C1s spectrum showed that the contents of C-O and C-OH groups sharply decreased and the content of C=O groups greatly increased. These changes were evidence of oxidation and the associated reaction between lignin and $[Ag(NH_3)_2]^+$.

Supplementary Notes 10: UV-vis Analysis of Lignin and Ag-Lignin NPs

Lignin and Ag-Lignin NPs suspension were recorded over the wavelength range from 200 to 800 nm using UV-vis spectrometer (TU-1901, Puxi, China) with a resolution of 0.5 nm. The NaOH solution was used as a blank.

The surface plasmon resonance of Ag-Lignin NPs was revealed by UV-vis spectra analysis. As shown in Supplementary Figure 9, with presence of Ag in the Ag-Lignin NPs, the enhancement of the quadrupole plasmon resonance was located in the region of 400 nm to 500 nm. With the increase of Ag content (in the samples of 0.05 NPs), the quadrupole plasmon resonant wavelength appeared approximately 360 nm.

Supplementary Notes 11: Preparation and characterization of the hydrogel

AA, pectin, Ag-Lignin NPs solution, APS, PEGDA, and water were poured into a beaker and stirred to prepare a homogeneous solution. Thereafter, the solution was injected into a reaction mold. Finally, the samples were placed in an N_2 atmosphere for 20 min at room temperature to obtain NPs-P-PAA hydrogels. The formulations of the hydrogels were

denoted as x NPs-P-PAA, where, x was the mass of $AgNO_3$. The composition of the various hydrogels is listed in Supplementary Table 5.

Supplementary Notes 12: Ag-Lignin NPs systems initiated the free radical polymerization of various monomers

The 0.05 NPs solution (4 mL), APS (5 wt.%), and water (4 mL) were poured into a beaker and stirred to prepare a homogeneous solution. Then, AA (2.7 mL), AM (2.6 g), or poly(ethylene glycol) diacrylate (2.6 mL) was added and the solution was injected into a reaction mold. Finally, the samples were placed in a N_2 atmosphere for 20 min at room temperature to obtain hydrogels. Our results indicated that the NPs could initiate polymerization of all of these monomers (Supplementary Figure 11). Thus, the current study provides a general method that could be used to prepare various hydrogels, not only PAA hydrogels, without UV irradiation or thermal assistance.

Supplementary Notes 13: XPS analysis of the hydrogels

The P-PAA and 0.03 NPs-P-PAA hydrogels were also analyzed by using XPS to reveal the catechol groups inner the hydrogel. The ratio of C-O(C-OH)/C=O was calculated to further confirm the presence of the catechol groups in the NPs-P-PAA hydrogel. The C-O(C-OH)/C=O ratio of the NPs-P-PAA hydrogel was 1.05 and higher than that of the P-PAA hydrogel, which indicated that C-O or C-OH (catechol) groups presented in the NPs-P-PAA hydrogel (Supplementary Table 6).

Supplementary Notes 14: The redox reaction between the NPs and excessive persulfate solution

The Ag-Lignin NPs were set as the working electrode, Ag/AgCl (KCl sat.) as the reference electrode, and Pt as the counter electrode. Solutions with different APS concentration (0, 0.1%, 0.5%, 1%, and 2%) and PAA (10%) were used as the electrolyte. CV measurements were performed at 5 mV s^{-1} between -0.20 and 0.80 V versus Ag/AgCl over four cycles.

A prominent redox peak at 0.10~0.20 V was observed (Supplementary Figure 13a). This corresponded to catechol oxidation and quinone reduction, which occurred at the same potential[5, 6]. These redox peaks still existed even with excessive persulfate (Supplementary Table 7, Supplementary Figure 13 b-f), which indicated that the stable redox reaction of Ag-Lignin NPs occurred even in excessive persulfate solution.

Supplementary Notes 15: Antioxidative abilities of the hydrogels tested by DPPH scavenging

The antioxidative abilities of the hydrogels were tested by measuring their capacities to scavenge stable DPPH free radicals using an established method with minor modifications[7]. The catechol groups of Ag-Lignin NPs in the hydrogels can scavenge DPPH free radicals[8]. First, a homogenate of the freezing-dried NPs-P-PAA hydrogels was prepared by grinding and dispersed in methanol (Supplementary Table 8). Next, DPPH (3.0 mL, 100 μM) was added and the mixture was incubated in dark for 30 min. Then, wavelength scanning was performed using a UV–Vis spectrophotometer (TU-1901, Puxi, China). The capacity of the NPs-P-PAA hydrogel to scavenge DPPH free radicals was evaluated using the following equation:

$$\text{DPPH scavenging}\ \% = \frac{A_B - A_H}{A_B} \times 100 \qquad \textbf{(2)}$$

where A_B is the absorption of the blank (DPPH + methanol) and A_H is the absorption of the hydrogel (DPPH + methanol + hydrogel). The reduction ability of bare Ag-Lignin NPs was also tested with the same method.

The results are shown in Supplementary Figure 14. The UV-vis peak of pure DPPH• appeared at 517 nm. After the pure P-PAA hydrogel was added, the intensity of the DPPH• peak did not decrease. By contrast, after the NPs-P-PAA hydrogel was added, the intensity of the DPPH• peak sharply decreased, which was attributed to the existence of catechol groups in the hydrogel. The Ag-Lignin NPs also exhibited good capacity to scavenge DPPH• (Supplementary Figure 14b). The percentages of DPPH• scavenged by the NPs-P-PAA hydrogel and NPs were 91.70% and 79.24%, respectively. These results indicate that both the NPs and NPs-P-PAA hydrogel have high reduction abilities.

Supplementary Notes 16: UV-vis analysis of a non-crosslinked polymeric system containing NPs

UV-vis analysis was conducted on a non-crosslinked polymeric system containing NPs to prove catechol groups in the polymeric system. The polymeric system without NPs was used as a control. First, 0.03 NPs-P-PAA and P-PAA polymeric systems without crosslinking was prepared. Second, they were diluted to 0.05 g mL^{-1} by deionized water. UV-vis tests was performed using an UV-vis spectrometer (TU-1901, Puxi, China). The UV-vis spectra showed a narrow absorption band at 280 nm (Supplementary Figure 15), which was assigned to the catechol groups of lignin[9]. These results confirm that catechol groups present in the hydrogels and they contribute to the adhesiveness, and also the redox reaction in the hydrogel.

Supplementary Notes 17: Morphology of the freeze-dried hydrogel

The fresh hydrogel samples were lyophilized at -80 °C. Then, the freeze-dried specimens were broken apart, and the inner morphology of the various hydrogels was observed by SEM (JSM 6390, JEOL, Japan).

Supplementary Notes 18: Element mapping of Ag distribution in the hydrogel

The 0.03 NPs-P-PAA hydrogels were lyophilized at -80 °C and then broken apart. The morphologies inside the hydrogels were observed by scanning electron microscopy element mapping using an energy dispersive spectrometry (JSM 6390, JEOL, Japan). The results indicated that Ag-Lignin NPs was distributed homogeneously inside the hydrogel (Supplementary Figure 17).

Supplementary Notes 19: Mechanical property testing

The hydrogels were molded into cylindrical specimens (D = 15 mm, H = 10 mm) for compression testing and rectangular specimens (L = 5 mm, W = 25 mm) for tensile testing. The mechanical property measurements of the hydrogels were performed using a universal testing machine (5567, Instron, America) with a 100 N load cell. The compressive tests were measured at a speed of 1 mm min^{-1} with a compression of 90%. The tensile test of the hydrogel was performed at an extension speed of 10 mm min^{-1}. The fracture energy was tested using a classical single-edge notch test according to a previous study[10]. The data of the tensile and compression tests were reported based on the average of four measurements.

Supplementary Notes 20: Adhesion tests

Tensile adhesion testing was performed to measure the adhesive strength of the PAA, P-PAA, NPs-P-PAA hydrogels to porcine skin. The hydrogels were applied to the surface of the

specimens with a bond area of 25 mm × 25 mm. The samples were pulled to failure using a universal testing machine (5567, Instron, USA) with a cross-head speed of 5 mm min^{-1} under ambient conditions. Adhesion-strip cyclic tests were also conducted to evaluate the effect of a cycle load on the adhesion strength of the hydrogels. The adhesive strength was calculated by the measured maximum load divided by the bond area.

Supplementary Notes 21: The effect of carboxyl groups of PAA on the adhesiveness of Ag-Lignin NPs hydrogel systems

Two types of hydrogels were prepared to prove the synergistic effect of the carboxyl groups of PAA and catechol groups of the Ag-Lignin NPs on the adhesiveness of the hydrogel. A gel with no carboxyl groups was prepared from polyacrylamide (PAM), Pectin and Ag-Lignin NPs, and gels with carboxyl groups were prepared from poly(acrylic acid-co-acrylamide) P(AA-co-AM), Pectin and Ag-Lignin NPs (Supplementary Table 9). As shown in Supplementary Figure 22, the adhesion strengths of NPs-P-PAM and NPs-P-P(AA-co-AM) hydrogels to porcine skin were 12 KPa and 15 KPa, respectively, and lower than that of the NPs-P-PAA hydrogel (25 KPa). These results prove that the adhesiveness of NPs-P-PAA is dependent on the synergistic effect of the carboxyl groups of PAA and catechol groups of Ag-Lignin NPs.

Supplementary Notes 22: Ag^+ release analysis

The antimicrobial activity of silver containing hydrogels was dependent on the release of Ag^+ from the hydrogel to the pathogenic environment. To evaluates the release of Ag^+, atomic absorption spectroscopy (AAS; PE, AA700, USA) was performed. Before examination, the release profiles of silver from the hydrogels were characterized in vitro by the all-change method in the PBS solution (pH 7.4). Here, 0.03 NPs-P-PAA hydrogels and 0.05 NPs-P-PAA

(30 μg sample^{-1}) hydrogels were immersed in the PBS solution (5 mL) and shaken (100 rpm) at 37 °C. At predetermined intervals (1 d, 3 d, 5 d, 7d, 14 d, and 21 d), the released PBS was collected and replaced by fresh PBS. The tests were repeated three times.

As shown in Figure 23, for the 0.05 and 0.03 NPs-P-PAA hydrogels, an initial burst release of silver was not observed. The Ag^+ exhibited a constant release during the release time. The amounts of Ag^+ released from the 0.05 and 0.03 NPs-P-PAA hydrogels were 0.566 mg L^{-1} and 0.537 mg L^{-1}, respectively, after 21 d of release. This low concentration of Ag^+ ions is safe for cells and tissues[11].

Supplementary Notes 23: Antibacterial activity in vitro

In order to investigate the antibacterial activity of the hydrogel, *Staphylococcus epidermidis* (*S. epidermidis*, ATCC6538, gram positive organism) and *Escherichia coli* (*E. Coli.*, ATCC8739, gram negative organism) were used for the tests refer to our previous study[12]. The hydrogel with NPs and pectin was used as a sample, and the hydrogel without NPs and pectin was used as a control. The antibacterial activity of five groups of samples (30 μg sample^{-1}), including PAA, P-PAA, and NPs-P-PAA with different concentrations of NPs, were tested by evaluating the inhibition of the bacterium *S. epidermidis* and *E. Coli.* Here, 100 μL of bacterial suspension (1×10^6 CFU mL^{-1}) was added to the samples. After 4 h, 900 μL of Luria-Bertani broth was added. Then, the samples were placed in an incubator at 37 °C with constant agitation. After 1 d, 200 μL of bacterial suspension was collected. The optical density (OD) of the suspension at 600 nm was measured using a micro plate reader (MQX200). The bactericidal ratios of the groups were calculated according to the following equation.

$$Bactercidal\ ratio(\%) = \frac{OD\ of\ contrastive\ groups - OD\ of\ extermental\ groups}{OD\ of\ contrastive\ groups} \times 100\% \quad \mathbf{(3)}$$

Supplementary Notes 24: Antibacterial activity in vivo

The antibacterial activities of the hydrogel were further confirmed in vivo in a rabbit model according to the previous study[12]. The P-PAA and NPs-P-PAA hydrogels were implanted subcutaneously on the back of rabbit, following which 1 mL of E. coli (10^5 cells mL^{-1}) was injected to the hydrogel site. The P-PAA hydrogels were treated as a positive control. In the days following the surgical operation, the animal activity and appearance of the wound was examined each day. The wound of the surgical site was observed. After 7 d post-surgery, the wound of the control group was surrounded with pustule, while the wound treated by the hydrogel exhibited healing. The surgical sites were harvested to examine infections and inflammatory reactions.

Supplementary Notes 25: Cell biocompatibility in vitro

NIH-3T3 fibroblast (SCSP-515, Stem Cell Bank, Chinese Academy of Sciences, Shanghai, China) cells in a growth phase were treated with trypsin and harvested. According to previous methods[12], the cells were suspended in the culture medium to obtain a cell density of 1×10^5 cells mL^{-1}, as counted by a hemocytometer. The cells were seeded on the hydrogels with a density of (5×10^4 cells) in the wells of the tissue culture plates and left undisturbed in an incubator for 3 h to allow for cell attachment. Then, an additional 1 mL of the Dulbecco's modified eagle medium (DMEM) supplemented with 10% fetal bovine serum (FBS) was added into each well. The cells were allowed to adhere and grow for 3 d and 5 d. The morphologies of the cells on the hydrogel surfaces were observed using a laser scanning confocal microscope (Leica, Germany).

The biocompatibility of the hydrogel and the cell proliferation was assessed by the 3-[4,5-dimethylthiazol-2-yl]-2,5-diphenyl tetrazolium bromide (MTT) assay. After 3 and

5 d of culture, the culture medium was replaced with DMEM supplemented with 100 μL of 3-(4,5-dimethylthiazol-2-yl)-2,5-diphenyltetrazolium bromide (MTT, 0.5 mg mL^{-1}) to each culture well, and the resulting mixture was incubated for 4 h at 37 °C. Subsequently, the MTT solution was removed, and 400 μL of dimethyl sulfoxide (DMSO) was added to dissolve the formazan crystals. The solution was incubated in a shaking incubator (37 °C, 1200 rpm) for an additional 15 min.

Supplementary Notes 26: Wound healing in vivo

Full skin wounds were created on the dorsal area of rats and treated with the P-PAA hydrogel, NPs-P-PAA hydrogels, and the epidermal growth factor (EGF)-loaded NPs-P-PAA hydrogels. The wounds treated without hydrogels were used as a control.

The surgical procedure was performed according to a previous study[13]. Five male Sprague Dawley (SD) rats weighing 180~220 g were used. After being anesthetized with pentobarbital (2 wt.%, 2 mL kg^{-1}), the dorsal area of the rats was depilated, and four full-thickness circular wounds (8 mm in diameter) were created on the upper back of each rat using a disposable 8-mm skin biopsy punch. On each rat, a blank wound without a hydrogel was used as a control. The P-PAA, NPs-P-PAA, and EGF-loaded hydrogels (EGF, 30 μg $sample^{-1}$, Shanghai Primegene Bio-Tech Co., Ltd.) were implanted on other wound sites of the rats. Five parallel specimens of each type of hydrogel were tested. The hydrogels were placed directly on the wounds. In addition, the wounds were covered with a 4 cm × 10 cm piece of a TegadermTM dressing (3 M, St. Paul, MN, USA) to keep the hydrogels in place and to protect them from infection. In the following 3 d, the rats were injected with penicillin to reduce the risk of infection. All animal procedures were performed in accordance with

protocols approved by the institutional animal ethics committee of the Southwest Jiaotong University and laboratory animal administration rules of China.

Supplementary References:

1. Milczarek G, Rebis T, Fabianska J. One-step synthesis of lignosulfonate-stabilized silver nanoparticles. *Colloid Surfaces B.* **105**, 335-341 (2013).
2. Bährle C, Nick TU, Bennati M, Jeschke G, Vogel F. High-Field Electron Paramagnetic Resonance and Density Functional Theory Study of Stable Organic Radicals in Lignin: Influence of the Extraction Process, Botanical Origin, and Protonation Reactions on the Radical g Tensor. *J Phy Chem A.* **119**, 6475-6482 (2015).
3. Richter AP*, et al.* An environmentally benign antimicrobial nanoparticle based on a silver-infused lignin core. *Nat Nanotech.* **10**, 817 (2015).
4. Milczarek G, Rebis T, Fabianska J, Biointerfaces SB. One-step synthesis of lignosulfonate-stabilized silver nanoparticles. *Colloid Surfaces B.* **105**, 335-341 (2013).
5. Milczarek GJL. Lignosulfonate-modified electrodes: electrochemical properties and electrocatalysis of NADH oxidation. *Langmuir* **25**, 10345-10353 (2009).
6. Movil-Cabrera O, Rodriguez-Silva A, Arroyo-Torres C, Staser JAJB, Bioenergy. Electrochemical conversion of lignin to useful chemicals. *Biomass Bioenerg.* **88**, 89-96 (2016).
7. Zhao X, Wu H, Guo B, Dong R, Qiu Y, Ma PXJB. Antibacterial anti-oxidant electroactive injectable hydrogel as self-healing wound dressing with hemostasis and adhesiveness for cutaneous wound healing. *Biomaterials* **122**, 34-47 (2017).
8. An L, Wang G, Jia H, Liu C, Sui W, Si CJIjobm. Fractionation of enzymatic hydrolysis lignin by sequential extraction for enhancing antioxidant performance. *Int J Biol Macromol.* **99**, 674-681 (2017).
9. Kai D, Chua YK, Jiang L, Owh C, Chan SY, Loh XJJRA. Dual functional anti-oxidant and SPF enhancing lignin-based copolymers as additives for personal and healthcare products. *RSC Adv.* **6**, 86420-86427 (2016).
10. Han L*, et al.* A Mussel-Inspired Conductive, Self-Adhesive, and Self-Healable Tough Hydrogel as Cell Stimulators and Implantable Bioelectronics. *Small* **13**,   (2017).

11. Xie CM*, et al.* Silver nanoparticles and growth factors incorporated hydroxyapatite coatings on metallic implant surfaces for enhancement of osteoinductivity and antibacterial properties. *ACS Appl Mater Inter.* **6**, 8580-8589 (2014).
12. Gan D*, et al.* Mussel-Inspired Contact-Active Antibacterial Hydrogel with High Cell Affinity, Toughness, and Recoverability. *Adv Funct Mater*. 1805964 (2018).
13. Han L*, et al.* Mussel-Inspired Adhesive and Tough Hydrogel Based on Nanoclay Confined Dopamine Polymerization. *ACS Nano* **11**, 2561 (2017).
